# Closing the loop in minimally supervised human–robot interaction: formative and summative feedback

**DOI:** 10.1038/s41598-024-60905-x

**Published:** 2024-05-08

**Authors:** Mayumi Mohan, Cara M. Nunez, Katherine J. Kuchenbecker

**Affiliations:** 1 Haptic Intelligence Department, Max Planck Institute for Intelligent Systems, 70569 Stuttgart, Germany; 2 Sibley School of Mechanical and Aerospace Engineering, Cornell University, Ithaca, 14853 USA

**Keywords:** Human behaviour, Biomedical engineering

## Abstract

Human instructors fluidly communicate with hand gestures, head and body movements, and facial expressions, but robots rarely leverage these complementary cues. A minimally supervised social robot with such skills could help people exercise and learn new activities. Thus, we investigated how nonverbal feedback from a humanoid robot affects human behavior. Inspired by the education literature, we evaluated formative feedback (real-time corrections) and summative feedback (post-task scores) for three distinct tasks: positioning in the room, mimicking the robot’s arm pose, and contacting the robot’s hands. Twenty-eight adults completed seventy-five 30-s-long trials with no explicit instructions or experimenter help. Motion-capture data analysis shows that both formative and summative feedback from the robot significantly aided user performance. Additionally, formative feedback improved task understanding. These results show the power of nonverbal cues based on human movement and the utility of viewing feedback through formative and summative lenses.

## Introduction

Gesture-based communication is as essential as its verbal counterpart for in-person human–human interaction^1^. For exercise and other motor-learning tasks, physical demonstrations and gestures become an even more important part of the exchange. Nonverbal communication is a critical component of interaction that assists with understanding^2^ and can sometimes overcome language barriers^3^. Thus, we believe that nonverbal robot behavior has the potential not only to influence user perception of a robot, but also to elicit behavioral responses and improve task performance^4^. Although there is extensive research on the detection of user gestures^5^, robot-generated gestures are only rarely studied in human–robot interaction (HRI) and are frequently combined with speech^6^. In particular, gesture-based interactions have mainly been studied for story-telling robots^7,8^, social interactions^9,10^, and educational activities for children^11^. However, nonverbal cues have not yet been thoroughly investigated for helping autonomous robots guide humans to perform physical tasks. This topic is especially timely given the rapid recent improvements in vision-based perception of humans^12,13^ and the increasing deployment of robots in everyday settings^14,15^.

Our interest centers on robotic exercise coaches that can be used in community rehabilitation settings. Regular physical activity is essential for maintaining wellness^16,17^. Exercise not only reduces the risk of developing chronic conditions^18^ but can also decrease stress^16^ and improve cardiovascular health^19^. Socially assistive robots^20^ are gaining recognition as robotic coaches that can motivate users to exercise^21^. Several cross-sectional studies have also found that people enjoy interacting with robotic exercise companions^22–25^. Such robots could help alleviate caregiver staff shortages^26–28^ by reducing healthcare workload and ensuring worker safety during pandemics^29,30^. These scenarios highlight the need for standalone robotic systems that can effectively coach human users in exercise, physical therapy, and other movement-based activities without close supervision by human experts.

Current research into minimally supervised systems for health-related HRI has primarily focused on home-based therapy for stroke rehabilitation^31–37^. Most of these systems are geared toward upper-limb rehabilitation and are based on joysticks^32^, orthoses^34,36^, or exoskeletons^33,35^, but researchers have also explored home-based lower-limb rehabilitation^37^. Some of these robotic rehabilitation systems can be used without caregiver supervision^32,34,36,37^, whereas the more sophisticated systems that enable multi-joint therapy require expert support^33^.

Social robots interact with users as independent social agents, not as mere devices. Here, the explicit idea of minimally supervised interaction is gaining popularity as the focus shifts to robots that can help users in everyday settings. Specifically, in-home socially assistive robots for children with autism^38–40^, in-home reading companions^41^, and care robots for older adults^42^ both at home and within care facilities^43^ have been extensively studied. Minimally supervised in-the-wild robot deployments have also been explored within schools^44^ and museums^45,46^ and as comedians^47^ and tour guides^48^. However, these existing socially assistive robots with minimal supervision have predominantly explored verbal^23,38,39,43,49–51^ and affective interactions^39,40,44^, with gesture-based exercise interactions restricted to closely supervised settings^23,24^.

This paper showcases the potential of nonverbal gesture-based feedback for autonomous social robots by making two major contributions. First, we provide an education-based paradigm for roboticists to consider when designing nonverbal feedback for minimally supervised HRI. Second, we show how to quantitatively analyze the effectiveness of nonverbal robot feedback. Existing research in gesture-based interaction has heavily focused on qualitative research methods such as surveys^7,8,11^ and thematic analysis^11,52^, which are time intensive and disconnected from the user’s physical performance of the task. We believe movement-based interactions such as exercising should primarily be evaluated via quantitative metrics that can also be used to strengthen and adapt robot feedback^53^.

To craft impactful feedback for minimally supervised contexts, we drew inspiration from the wealth of insights by education researchers^54,55^. Educators adeptly employ two common types of assessments, formative and summative, across diverse domains, such as language learning^56^, accounting courses^57^, and online classrooms^58^, demonstrating the versatility of these strategies in fostering student learning. Furthermore, these assessments help both children^59^ and adults^60^. Formative assessment is performed during a course of instruction; it is meant to help identify the student’s strengths and weaknesses and also improve autonomy, responsible learning, and self-evaluation skills^55,61^. Additionally, formative feedback supports educators by helping them devise subsequent instruction strategies^55^. On the other hand, an assessment that is administered at the end of a unit of instruction and categorizes student performance^55,61^ is called summative assessment.

Deriving from these ideas, we propose that nonverbal robot feedback about human activities should be divided into two types based on formative and summative assessments. Thus, we define the following two feedback strategies for a socially assistive robot: *Formative feedback* is provided during the interaction and aims to improve the user’s competence at the task, and *summative feedback* gives the user an overall assessment of their performance at the end of a particular task. We are also guided by educational principles in designing our feedback, incorporating concepts such as dual-coding theory^62^: information is more likely to be remembered when presented with both verbal and nonverbal symbolism. Our robot, thus, employs a range of multimodal techniques, including auditory cues, facial expressions, and gestures. To evaluate the two proposed feedback types, we draw the following hypotheses from education and extend them to the domain of minimally supervised nonverbal human–robot interaction.

### H1

Formative and summative feedback independently improve user performance^54,63^.

### H2

Formative feedback promotes user understanding of the task^54,55^.

Experimental support for these hypotheses, as provided by our rigorous user study, indicates that social robots can better support humans in performing physical exercise by providing nonverbal performance feedback both during and after each activity. Though more complex to implement, formative feedback is particularly powerful at facilitating successful interactions because it improves user understanding. Given their impact in this minimally supervised exercise scenario, these types of robot behaviors combining real-time human observation with gestural communication would likely also benefit autonomous robots working with people in other challenging settings.

## Results

We examined our hypotheses quantitatively, evaluating the dynamic and unstructured behavior of naive users in response to formative and summative feedback delivered by a robot during minimally supervised activity sessions. The study took place in our Robot Interaction Studio^64^, a room containing an upper-body humanoid robot (Baxter^65^) and a camera-based markerless motion-capture system (Captury Live^66^) that tracks the user’s pose in real time. The robot’s behavior was carefully designed to convey the task cues and both types of feedback clearly and consistently via iterative pilot testing.

**Figure 1:**
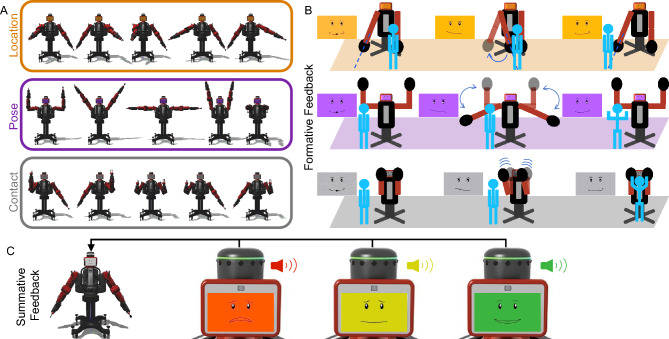
**(A)** The three cue categories (location, pose, and contact), which each had five variants. **(B)** The formative feedback adopted by the robot for each cue type. The robot performs the gesture associated with the cue and makes an expression to show that it is waiting for the user (left). After several seconds of the user not doing the desired action, the robot displays a disappointed smirk and performs the corrective gesture of formative feedback (middle), which is customized for each cue. Conversely, the robot shows a happy face while the user executes the task correctly (right). If the user stops doing the desired action, the robot shows the waiting face again and will make periodic corrective gestures until the user corrects their action or the trial ends. The location cue (top row) is modeled after pointing gestures. The robot points to a location on the ground within the Robot Interaction Studio for the user to move to. For formative feedback, the robot points to the user’s current incorrect location and then to the desired location. The five variants of the pose cue (second row) show the symmetric arm pose the user should imitate. For formative feedback, the robot shows an intermediate arm pose between the desired pose and a neutral pose based on the magnitude of the user’s current pose error. Finally, for the contact cue (third row), the robot signals for contact by holding up one or two of its hands similar to a high five. For formative feedback, the robot wiggles its forearm (elbow joint) for a duration proportional to the current distance from the user’s hands to the robot’s offered hand(s). **(C)** The way in which the robot conveyed summative feedback at the end of each trial, including presentation of a red, yellow, or green face with an animated facial expression and a matching auditory cue.

We conducted a mixed-design study that had two between-subjects factors (formative and summative feedback, each with two levels) and one within-subject factor (cue type with three levels). The study included 28 participants with 7 participants in each group. Each study participant tried to interpret the same three gesture-based robot cues under one of four feedback configurations: no feedback (fs), only formative feedback (Fs), only summative feedback (fS), or both types of feedback (FS). Participants were told to do their best to interpret the robot’s cues to perform the tasks; the experimenter never explained any aspect of the robot’s behavior and answered no questions. Figure 1 shows the three gesture-based cue types experienced by all participants, their five variants, their associated types of formative feedback, and how summative feedback was conveyed across tasks. For all five variants of the three cue types (Fig. 1A), the robot gestures to try to make the user perform a specific task that is never verbally explained. For the location cue, the participant is supposed to go to the indicated location and stand there for the entire trial. For the pose cue, the participant should mimic and hold the robot’s arm posture. Finally, for the contact cue, the participant should constantly touch the robot’s offered hand(s). Participants experienced 25 30-s-long trials of each type of cue, always presented in the repeating order location cue, pose cue, and contact cue for a total of 75 trials. The Supplementary Videos show one sample robot instruction for each cue type (Video SV1) and six sample trials for a participant in the FS condition (Video SV2).

**Figure 2:**
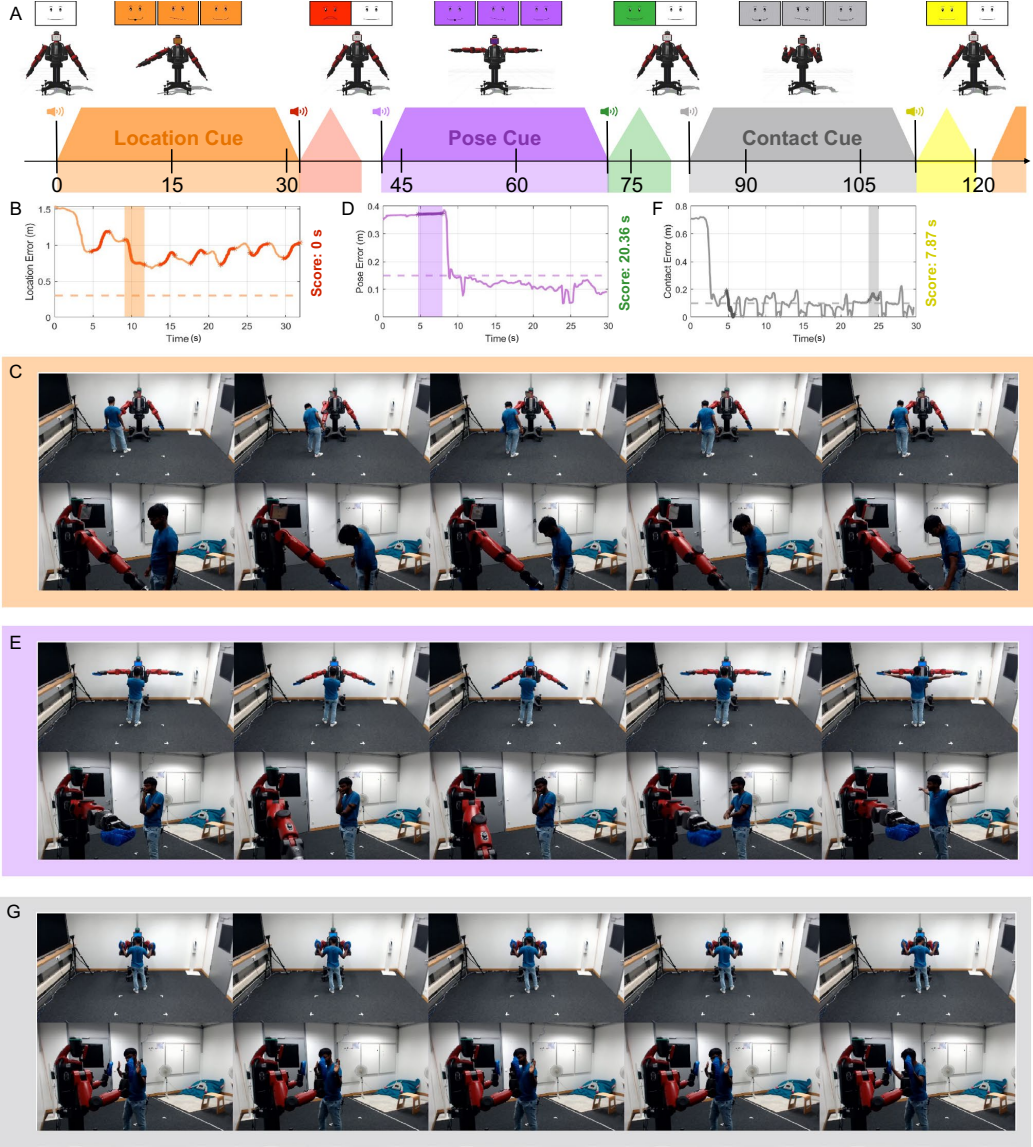
**(A)** Sample timeline of three successive cues when both formative and summative feedback were provided (FS); the graphics above the timeline depict the robot facial expressions^67^ and arm gestures. Three expressions are associated with each cue type. The first is the waiting face shown to participants in all four conditions; the robot displays this face while waiting for the participant to perform the task. The other two are tied to formative feedback: a positive facial expression shows while the user is doing the desired action, and a negative facial expression displays during each corrective gesture. At the end of each trial, the robot displays the summative feedback for several seconds before returning to its neutral pose. **(B)** User error over time for a sample trial of the location cue. The participant did not figure out this task and thus never achieved an error below the threshold (indicated with a dotted line), resulting in a final score of 0 s. The highlights in the line plot depict the seven instances of formative feedback. **(C)** Screenshots from one instance of formative feedback for the location cue. The user incorrectly attempts to hold the robot’s hand; the robot points to the user’s feet and then points to the correct location. **(D)** User error over time for a sample trial of the pose cue with only one instance of formative feedback. **(E)** Example formative feedback instance for the pose cue. The user starts out not performing the task. The robot provides formative feedback by moving its arms based on the error metric. The user then mimics the robot. **(F)** User error over time for a sample trial of the contact cue. The user repeatedly touches the robot’s hand and then moves their hand back, causing oscillations in the error signal. Formative feedback is provided only when the user has not performed the task correctly for at least 5 s, which occurs twice. **(G)** Example formative feedback for the contact cue. The user can be seen mimicking the robot. The robot then performs a hand wiggle as formative feedback, and the user touches the robot’s palm. All sample trials shown in this figure were taken from a participant who received both types of feedback. This participant provided informed consent to allow videos associated with this research to be shown publicly.

Figure 2 shows how the robot behaved over time during the study, highlighting three successive trials from one participant with plots of their error over time and sample video frames. The markerless motion-capture system provided full-body tracking of the user as they interacted with the robot^64^. These real-time measurements were used to drive the formative feedback provided by the robot during trials for Fs and FS participants (Fig. 1B). In these conditions, the robot provided additional assistance via facial expressions and arm gestures during each 30-s trial to help the user identify the solution. The formative feedback does not explicitly provide the solution to the user; rather, it nudges them toward it. Even if a participant discovers an action results in positive formative feedback, they must figure out which aspect of that action is essential and that they need to continue doing it. The motion-capture data was also used to calculate the score achieved in each trial by counting the amount of time the user performed the desired task with less than a specified error threshold; the robot presented a binned version of this summative score to participants in the fS and FS groups after each trial (Fig. 1C). Participant heart rate (Fig. S8) and facial expression were collected throughout the study, and an extensive questionnaire was completed at the end (Supplementary Materials Sect. 2). The “Materials and methods” section provide a detailed explanation of the study and cue design. The results were evaluated in terms of participant performance, cue comprehension, and activity.

### Participant performance

**Figure 3:**
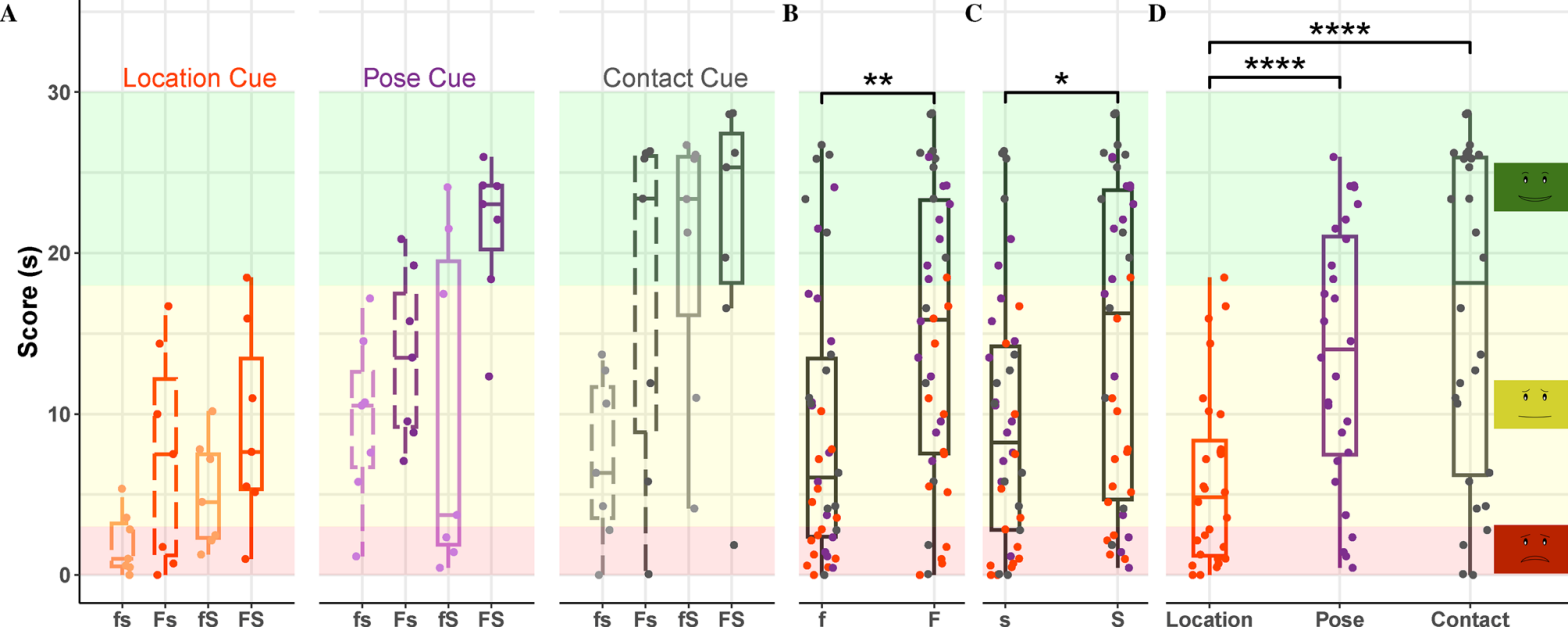
**(A)** Boxplots of mean participant score for each condition (no feedback (fs), only formative feedback (Fs), only summative feedback (fS), or both types of feedback (FS)) and cue types. Higher scores are better, and the maximum is 30 s; the background colors depict the three levels of summative feedback. Participants performed better when the robot provided either type of feedback. **(B)** Boxplots of mean participant scores separated by level of formative feedback. Formative feedback significantly increased participant scores. **(C)** Boxplots of mean participant scores separated by level of summative feedback. Summative feedback also significantly increased participant scores. **(D)** Boxplots of mean participant scores separated by cue type. Participants earned higher scores in the pose cue and the contact cue compared to the location cue. For all boxplots, the dots mark the data points (one per participant per cue), the central line shows the median, the box shows the lower and upper quartiles, and the whiskers show the range up to 1.5 times the interquartile range. Significance notation: *p < 0.05, **p < 0.01, and ***p < 0.0001.

The main outcome measure is the score the user earned in each trial, calculated as the duration of time they performed the desired action with an error less than the defined threshold. A three-way non-parametric Aligned Rank Transform (ART) ANOVA^68^ was performed to evaluate the effects of formative feedback, summative feedback, and cue type on the user’s average score (Fig. 3), which could range from 0 to 30 s. Importantly, the main effects of formative feedback $(F(1,24) = 10, p = 0.0036, \eta _p^2 = 0.30)$, summative feedback $(F(1,24) = 7.4, p = 0.012, \eta _p^2 = 0.24)$, and cue type $(F(2,48) = 24, p = 6\times 10^{-8}, \eta _p^2 = 0.50)$ were all significant. None of the interactions were significant, but there was a trending three-way interaction between formative feedback, summative feedback, and cue type $(F(2,48) = 3.1, p = 0.052, \eta _p^2 = 0.12)$. Post-hoc analyses revealed that the presence of either formative feedback $(t(24) = 3.2, p = 0.0036, d = 1)$ or summative feedback $(t(24) = 2.7, p = 0.012, d = 0.94)$ significantly improved user performance. Furthermore, participants performed better in the pose cue $(t(48) = 5, p = 2.4\times 10^{-5}, d = 1.3)$ and the contact cue $(t(48) = 6.6, p = 7.7\times 10^{-8}, d = 1.8)$ than the location cue.

We also examined participant performance over the course of the study via three additional metrics: mean time to first perform the desired action within a trial, first trial number to reach at least a middle (yellow) summative score, and first trial to reach a high (green) summative score (Supplementary Materials Sect. 11). Formative feedback significantly reduced the time users needed to begin performing the target action within a trial and the number of trials needed to obtain a high summative score. Though not significant, summative feedback also helped all three metrics. These results indicate that formative feedback in particular aided participant learning over time.

### Participant cue comprehension

**Figure 4:**
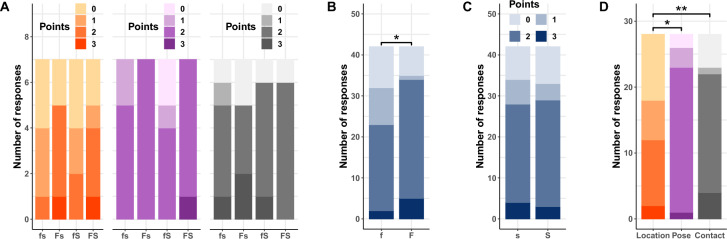
**(A)** Grades of participant comprehension of each task cue based on structured grading of written explanations (seven participants in each feedback condition). **(B)** Grades separated by level of formative feedback (fourteen participants in each formative feedback level with responses for all three cue types). Formative feedback significantly improved understanding, with more participants scoring 2 or 3 points out of 3. **(C)** Grades separated by level of summative feedback (fourteen participants in each summative feedback level with responses for all three cue types). Summative feedback did not affect user understanding. **(D)** Grades separated by cue type (all 28 participants for each cue type). Participants understood the pose and contact cues better than the location cue. Significance notation: $\star ~p<0.05$ and $\star \star ~p<0.01$.

Another important outcome measure is how well the participants understood the three task cues. As shown in Fig. 4A, we used an integer scale from 0 to 3 to grade their post-study written explanations of what the robot wanted them to do for each cue type (Supplementary Materials Sect. 3). Interestingly, there were significant main effects of formative feedback $(F(1,24) = 4.81, p = 0.038, \eta _p^2 = 0.17)$ and cue type $(F(2,48) = 6.8, p = 0.0025, \eta _p^2 = 0.22)$ on the cue comprehension grade (Fig. 4B,D). In contrast, as shown in Fig. 4C, the main effect of summative feedback was not significant $(F(1,24) = 0.35, p = 0.56, \eta _p^2 = 0.01)$. The grade was significantly higher for participants who received formative feedback $(t(24) = 2.2, p = 0.038, d = 0.56)$, and it was higher for the pose cue $(t(48) = 3, p = 0.013, d = 0.8)$ and the contact cue $(t(48) = 3.4, p = 0.0045, d = 0.9)$ in comparison to the location cue. Additional self-reported metrics related to cue understanding can be seen in the supplementary materials (Figs. S6, S7). Finally, the lack of explicit task instructions, the experimenter’s refusal to answer questions, and the resulting uncertainty about what to do tended to frustrate participants, as evidenced by their slightly more negative perception of robots after the study (Supplementary Materials Sect. 4). Feedback, however, positively affected the participants’ feelings of self-efficacy (Fig. S3D). Interestingly, although participants experienced negativity and frustration during the study, both formative and summative feedback enhanced their performance and understanding.

### Participant activity

**Figure 5:**
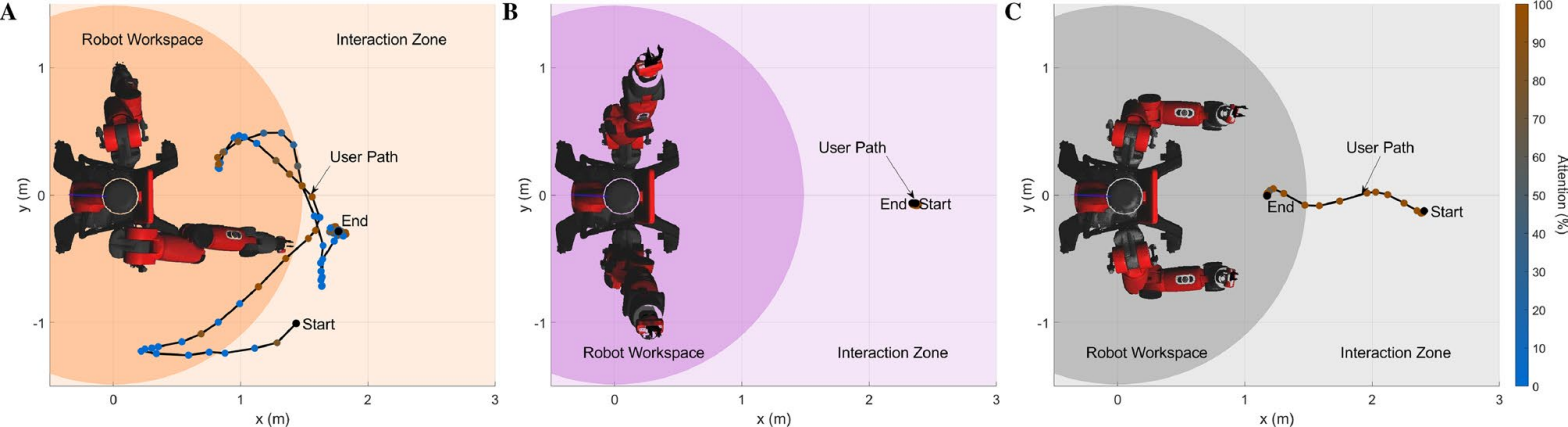
**(A)** Overhead view of a sample location cue trial from the condition with both formative and summative feedback (FS). The user walks around the room, leaving and entering the robot’s workspace and occasionally looking at the robot’s face. **(B)** Sample pose cue trial. The user stands in one position to perform the task. **(C)** Sample trial of contact cue. The user walks into the robot’s workspace to touch its hands.

**Figure 6:**
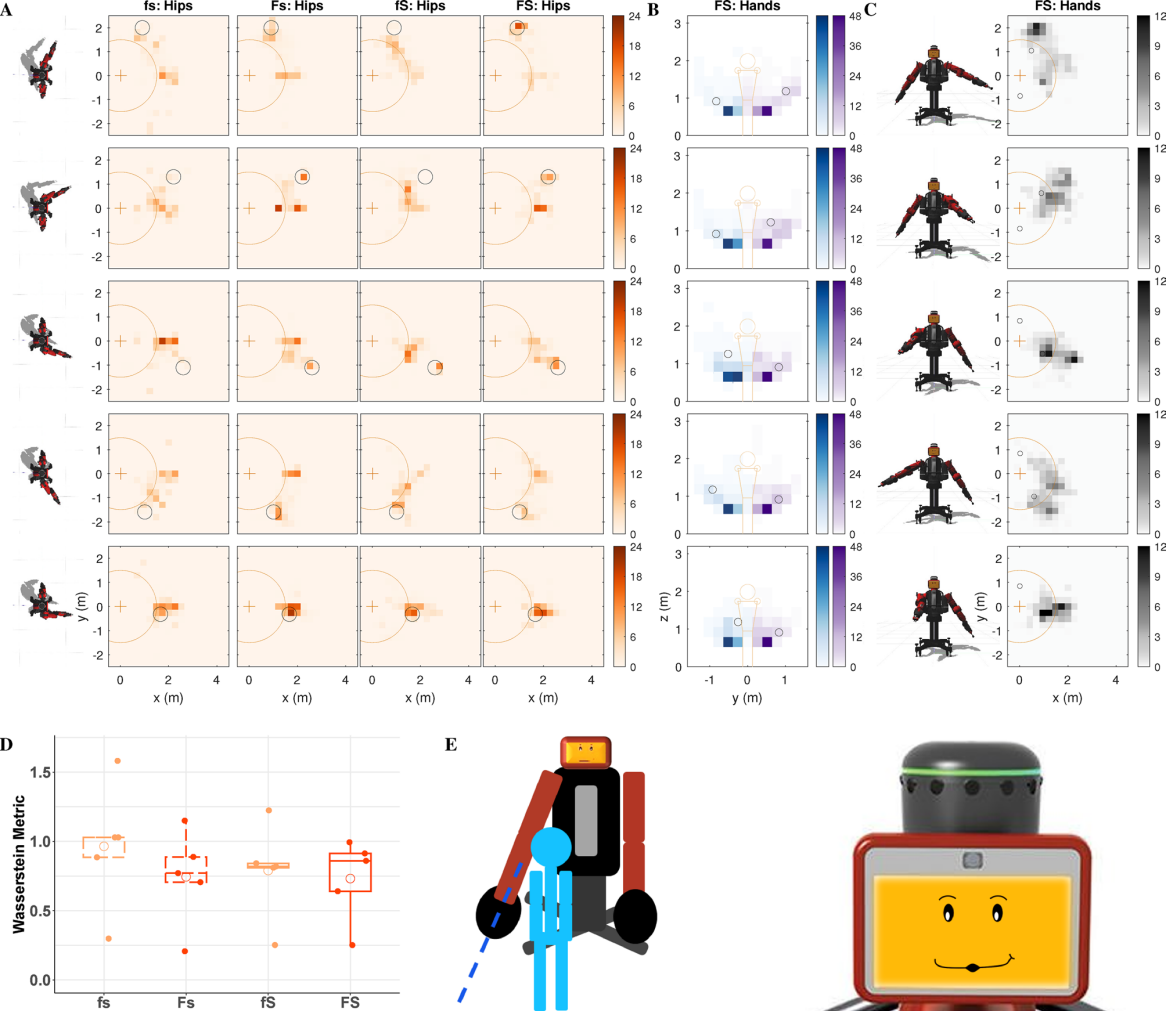
User activities across different feedback conditions of the location cue. **(A)** Heatmap visualizations of user position in the workspace of the Robot Interaction Studio. Across all conditions, users can be seen spending time at various locations around the room. When feedback is provided, the users tended to spend more time near the target position depicted by the black circle. **(B)** Heatmap visualizations of the front view of the user’s hands for the FS condition. Participants sometimes mimicked the robot’s arm pose, depicted by the black circles, and they usually kept their arms at their sides as they walked to and stood at the correct location. **(C)** Heatmap visualizations of the overhead view of the user’s closer hand in the horizontal plane for the FS condition. This heatmap resembles the FS condition of the user position heatmap because users often walked to the desired location. **(D)** Boxplot of the Wasserstein metric representing how much participant position in the room deviated from the correct location. **(E)** A sample location cue and the face associated with the cue seen by participants across all conditions.

**Figure 7:**
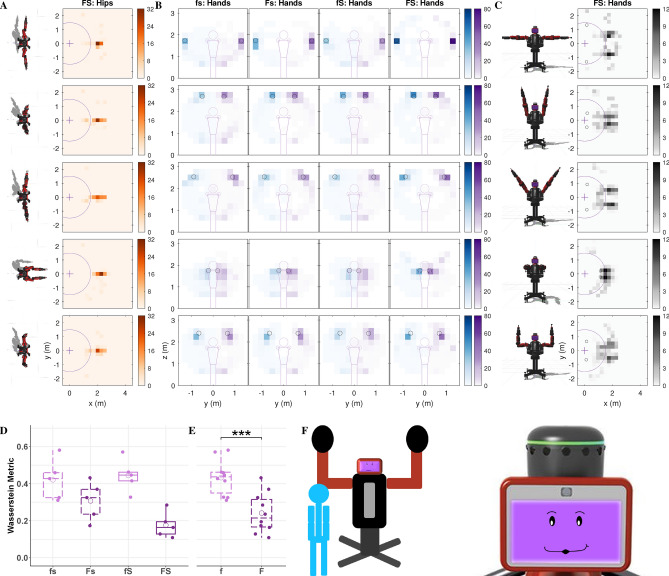
User activities across different feedback conditions of the pose cue. **(A)** Heatmap visualizations of user position in the workspace of the Robot Interaction Studio for the FS condition. Users tend to stand in front of the robot. **(B)** Heatmap visualizations of the front view of the user’s hand positions. In general, participants across all conditions figured out the desired behavior of mimicking the robot’s arm pose for this cue. However, their imitation of the pose was better when feedback was provided. **(C)** Heatmap visualizations of the overhead view of the user’s hand(s) in the horizontal plane for the condition where both types of feedback were provided. Since participants generally stood in front of the robot, and since all poses are symmetric, one can see both the left and right hands. **(D)** Boxplot of the Wasserstein metric representing how much participant hand positions deviated from the correct hand positions. The data presented here is the grand mean of the Wasserstein metric between actual and desired hand positions for both left and right hands. We look at the front view in this case, since the focus is on the arm pose. **(E)** Wasserstein metric separated by level of formative feedback. The presence of formative feedback had a positive impact on pose cue performance. Participants mimicked the robot more accurately when they had formative feedback to help correct their pose. **(F)** A sample pose cue and the face associated with the cue seen by participants across all conditions.

**Figure 8:**
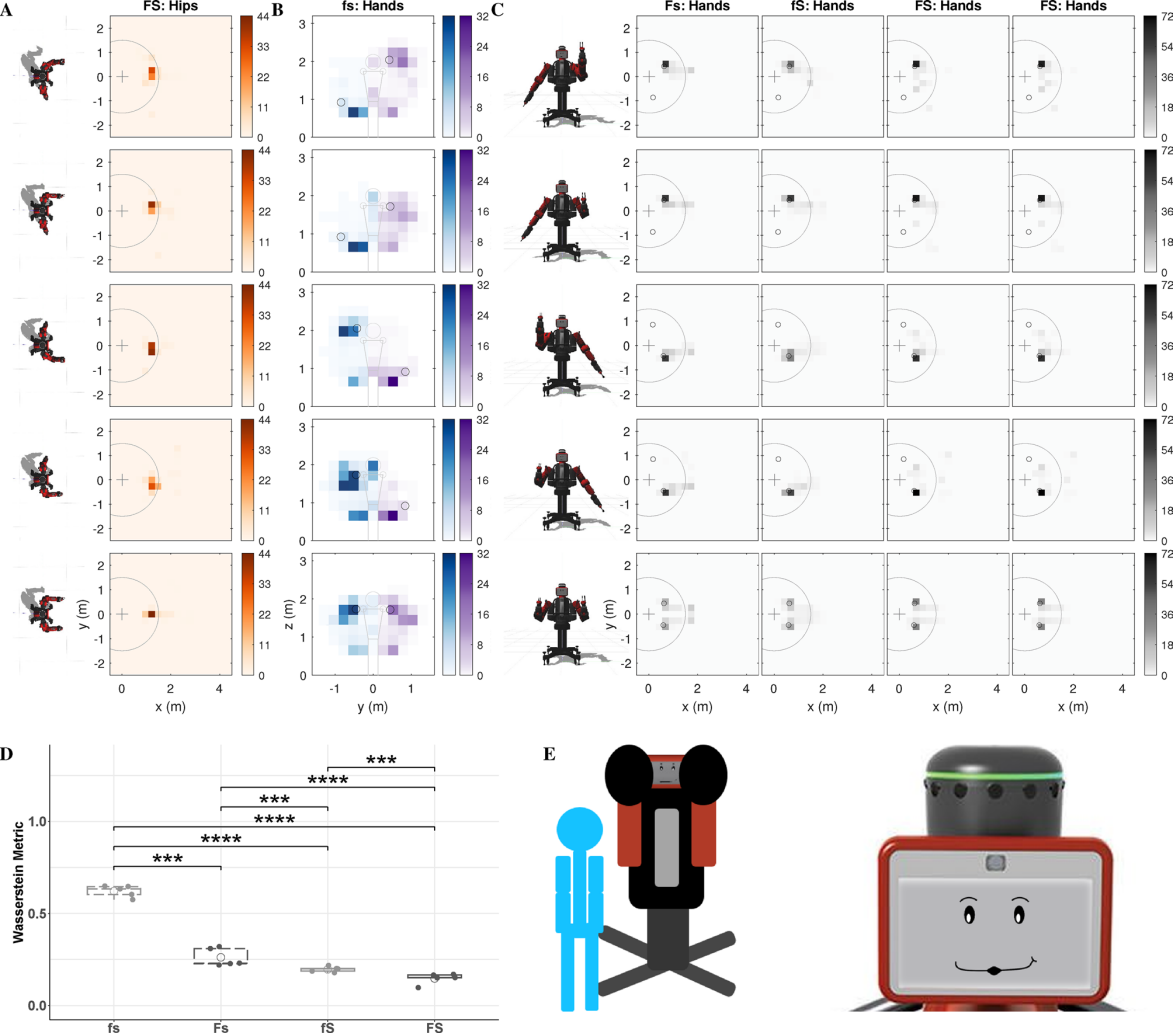
User activities across different feedback conditions of the contact cue. **(A)** Heatmap visualizations of user position in the room for the FS condition. Participants generally stood directly in front of the robot to perform this task. **(B)** Heatmap visualization of the front view of the user’s hands relative to their body for the FS condition. From this angle, it appears as though the user is mimicking the robot’s pose since they often hold their arms similarly to touch the robot’s hand(s). **(C)** Heatmap visualizations of an overhead view of the user’s hand(s) for all conditions. The first four rows show the position of the user hand that was closest to the robot’s offered end-effector; both robot hands were presented in the fifth variant of this cue, so that row depicts both hands of the user. This task cue was the easiest of all cues. Almost all participants figured out the solution to this cue when feedback was provided: they entered the robot’s workspace and touched the robot’s offered hand(s). **(D)** Boxplot of the Wasserstein metric representing how much participant hand positions in the room deviated from the correct hand positions. There was a significant interaction between formative and summative feedback. All feedback conditions were significantly different from the fs condition. The FS condition was significantly better than all other conditions, and only formative feedback (Fs) was better than only summative feedback (fS). **(E)** A sample contact cue and the face associated with the cue seen by participants across all conditions.

The effectiveness of formative and summative feedback can be understood by examining the actions participants performed as they tried to figure out the tasks cued by the robot in each feedback condition. As shown in Fig. 5 and Supplementary Fig. S9, we evaluated their behavior in terms of the distance traveled within the room, the level of attention to the robot’s face, and the amount of time spent within the robot’s reachable workspace. Furthermore, heatmap visualizations were created to show the cumulative spatial distribution of the user’s position in the room, their hand positions relative to their body, and their hand positions in the room across all trials of each of the five variants of the location cue (Fig. 6), the pose cue (Fig. 7), and the contact cue (Fig. 8). For these visualizations, the planar space under consideration was discretized into a grid, and the color shows the percentage of total trial time users spent in each grid cell. The resulting heatmaps provide a dense overview of what transpired during the study; for conciseness, we show all four feedback conditions for the heatmap type most relevant to each cue and only the condition with both formative and summative feedback (FS) for the other two heatmaps. The supplementary materials (Figs. S10–S12) show all three heatmap types for all four feedback conditions across all three task cues.

To test whether formative and/or summative feedback changed how users moved for each task, we quantitatively analyzed the heatmaps by calculating the Wasserstein metric^69^ between each measured distribution and a normal distribution representing correct user behavior, which was centered on the target position and dispersed by the acceptable threshold. Heatmaps that are closer to the desired user behavior have a smaller Wasserstein metric.

#### Location cue

The desired behavior was standing where the robot pointed. In general, the presence of formative feedback helped guide participants toward the correct position (Fig. 6A). Although this task was difficult for most participants (low scores, high Wasserstein metrics), two resourceful individuals figured out most variants of this cue with only summative feedback. Participants would sometimes try mimicking the robot’s arm poses, as seen in the blue and purple heatmaps (Fig. 6B). They frequently walked to and grasped the robot’s pointing hand, as seen in the gray user-hand-position heatmap in Fig. 6C, which was similar to the desired action. Despite these visual trends, a two-way Aligned Rank Transform (ART)^68^ ANOVA evaluating the Wasserstein metric showed that neither type of feedback had a significant effect on how close users stood to the target location for this cue. A three-way ART ANOVA showed that the total distance travelled by the participant per trial $(F(2,48) = 5.4, p = 0.0076, \eta _p^2 = 0.18)$ had a statistically significant two-way interaction between formative feedback and the type of cue (Fig. S9A); however, further analysis of this interaction did not reveal any significant simple effects at $\alpha =0.05$.

#### Pose cue

Participants typically mimicked the robot’s cues across all feedback conditions, as evident from the high concentration of blue and purple near the desired hand positions in the heatmaps (Fig. 7B). A two-way ART ANOVA on the Wasserstein metric based on the heatmap data revealed a statistically significant main effect of formative feedback on the hand position $(F(1,16) = 22, p = 0.00023, \eta _p^2 = 0.58)$; formative feedback significantly reduced the Wasserstein metric $(t(16) = 4.7, p = 0.00023, d = 2.1)$, indicating an improvement in replicating the robot’s movement. The overhead heatmaps show that participants tended to stand in the center of the room to perform the desired pose; accordingly, visual inspection of the pose cue data in Fig. S9A shows a statistically non-significant trend of formative feedback reducing the distance the user traveled $(F(1,26) = 5.4, p = 0.084, \eta _p^2 = 0.17)$. Interestingly, users looked at the robot’s face more often during the pose cue (Fig. S9B,C), as shown by a significant effect of cue type on attention $(F(2,48) = 20, p = 5.1\times 10^{-7}, \eta _p^2 = 0.45)$ with post-hoc analyses indicating that participants paid more attention to the robot for this cue in comparison to the contact cue $(t(48) = 4.6, p = 9.7\times 10^{-5}, d = 1.2)$ and the location cue $(t(48) = 6, p = 6.4\times 10^{-7}, d = 1.6)$. There is also a statistically trending effect of formative feedback on attention $(F(1,24) = 3.78, p = 0.06, \eta _p = 0.14)$. Visual inspection of these data hint that formative feedback may increase attention to the robot for the pose cue, though this trend did not reach significance.

#### Contact cue

The desired behavior for the contact cue was to touch the robot’s hands and maintain contact for the duration of the trial. Occasionally, participants incorrectly mimicked the robot’s pose for cues of this type. Many participants figured out this cue even without feedback. However, either type of feedback helped participants identify the correct behavior, as characterized by the top view of the hand positions (Fig. 8C), which shows darkly colored tiles overlapping with the correct hand positions. The two-way ART ANOVA on the Wasserstein metric showed a two-way interaction between summative and formative feedback $(F(1,16) = 50, p = 2.6\times 10^{-6}, \eta _p^2 = 0.76)$. The presence of only formative $(t(16) = 5, p = 0.00079, d = 1.6)$, only summative $(t(16) = 10, p = 1.6\times 10^{-7}, d = 3.2)$, or combined feedback $(t(16) = 15, p = 4.6\times 10^{-10}, d = 4.7)$ significantly improved the user’s ability to perform the task. Additionally, participant performance was better when only formative feedback was present when compared to pure summative feedback $(t(16) = 5, p = 0.00079, d = 1.6)$. The presence of both types of feedback led to a significantly better performance than providing only formative $(t(16) = 10, p = 1.6\times 10^{-7}, d = 3.2)$ or only summative feedback $(t(16) = 5, p = 0.00079, d = 1.6)$.

Participants largely figured out that they needed to enter the robot’s reachable workspace to correctly perform this cue (Fig. S9D,E). A three-way ART ANOVA on the time spent in the robot’s workspace showed that both formative feedback $(F(2,48) = 8.2, p = 0.00089, \eta _p^2 = 0.25)$ and summative feedback $(F(2,48) = 5.3, p = 0.0086, \eta _p^2 = 0.18)$ had significant two-way interactions with cue type. Post-hoc analyses revealed that the simple main effect of summative feedback $(F(1,26) = 13, p = 0.0033, \eta _p^2 = 0.34)$ was significant only for the contact cue, with further analysis indicating that the time spent in the workspace was significantly higher when summative feedback was present $(t(26) = 3.7, p = 0.0011, d = 1.4)$. Despite a statistically significant interaction between formative feedback and cue type $(F(2,48) = 8.2, p = 0.00089, \eta _p^2 = 0.25)$, post-hoc analyses did not reveal additional significant results.

## Discussion

For robots to interact effectively outside laboratory environments, they must be able to communicate with and engage users with minimal supervision from human experts. The presented study shows how an autonomous exercise robot can use multimodal nonverbal cues to convey task instructions, correct user behavior in real time, and communicate overall assessments of performance. Since education researchers have repeatedly proven the merits of both formative and summative assessments^54,70^, we tested these two kinds of feedback in a minimally supervised setting where users had to try to figure out three types of task cues based only on the actions of the robot. The results resoundingly support our hypotheses, showing that these time-tested feedback mechanisms based on human behavior can enable seamless user interactions with autonomous robots; they also open several interesting discussion points for future work.

Formative assessments are ongoing evaluations that support learners in improving their performance and increasing their understanding of the material^54,63,71^. As predicted by H1 and H2, the nonverbal formative actions our robot used to respond to user actions in real time helped in both of these ways: Formative feedback led to significantly higher average trial scores (Fig. 3B,H1) and significantly better post-study explanations of the tasks (Fig. 4B,H2). Formative assessments have been used within the classroom to improve both teaching strategies and student learning^59^. Our results suggest that robotic coaches can similarly leverage the powerful paradigm of formative assessment; providing formative feedback that responds to what the user is currently doing provides highly useful granular information about correct and incorrect actions. Achieving the performance and understanding benefits of formative feedback requires a robotic system that measures user activity in real time and rapidly outputs corrective feedback when errors are detected. Effective formative feedback lies at the core of providing personalized support, facilitating skill development, and promoting user engagement.

Summative feedback, on the other hand, has repeatedly been shown to aid learner performance^56,71^ but is not thought to aid comprehension. Indeed, as predicted by H1, the summative feedback provided by our robot significantly increased average trial scores (Fig. 3C); it had no impact on the participants’ ability to describe the tasks (Fig. 4C). These findings match well with existing education literature that states that summative feedback communicates the current state of the user’s knowledge, whereas formative feedback supports the process of acquiring knowledge and skills over time^72^. Summative feedback helped participants achieve higher task scores, but it did not improve their ability to articulate what they were supposed to do. Formative feedback, on the other hand, supported participants’ learning process by showing them whether what they were doing was correct at each instant in time. Advantageously, summative feedback has lower technical requirements than formative feedback, as it presents one summarized score at the end of an activity, with no real-time reactions. Robots can combine formative and summative feedback, since the benefits of these two types of feedback are largely independent.

Since we wanted to study the impact of formative and summative feedback, we purposefully designed the tasks and robot cues to present a challenge for users so their learning process would depend on their assigned feedback condition. Our three resulting task cues can be categorized in terms of ease of understanding (Fig. 4D) and ease of performance (Fig. 3D). The location cue was more difficult to understand than the other cues; since it was rarely understood, it was also rarely performed correctly, even though standing in a particular location is an easy activity. Most users (82.14%) correctly understood that they should copy the robot’s pose in the pose cue, though the selected error tolerance was difficult for some individuals to maintain for 30 s. The contact cues were easy to understand and easy to perform. Most participants (78.57%) figured out that they needed to touch the robot’s hand and could easily execute this action.

Summative feedback was consistently conveyed across all tasks, and participants seemed to understand its meaning through the redundant face colors, facial expressions, and sounds. The influence of summative feedback might have been greater if it had been designed to present the score with more granularity. The facial expressions that the robot used to provide formative feedback were also clearly understood and valued by participants; since they were more complex and unusual, the formative gestures often took several trials for participants to decipher. Simultaneously, it is useful to note that the formative gestures the robot executed for the three task cues provided different amounts of information: error magnitude and direction for location, error magnitude for pose, and quantized error magnitude for contact, roughly matching task difficulty.

The heatmaps created from the motion-capture data offer a dense overview of user activity during each task. The values of the corresponding Wasserstein metrics reiterate the relative challenge of the three cue types, with the location cue (Fig. 6D) being more difficult than the pose (Fig. 7D) and contact (Fig. 8D) cues. Though visible by eye (Fig. 6A), the distribution shifts caused by feedback in the location cue did not reach statistical significance. This finding may mean that the benefits of feedback are limited when a task is difficult to understand, or that our implementation of formative feedback for this cue was not clear to participants. Formative feedback was particularly useful for inducing users to move their hands closer to the target locations in the pose cue (Fig. 7B,D), whereas both types of feedback independently helped users solve the contact cue (Fig. 8C,D). Robotic formative feedback may help with a wider range of tasks than summative feedback, likely due to its support of task understanding.

Formative and summative feedback should be synergistic in nature and feed into one another^59,73,74^; formative assessment fosters learning while summative assessment validates it^71,75^. Each type of feedback independently increased the score of the user (Fig. 3B,C), strongly supporting H1. H2 was also strongly supported: Only formative feedback enhanced user understanding of the task (Fig. 4B,C). These observations are fully consistent with the education literature, which states that feedback in general improves performance^54^ and formative feedback promotes understanding of the task^54,55^. Minimally supervised robots, especially comprehensive exercise coach robots, can more effectively support community rehabilitation by providing both types of feedback. When present simultaneously, these types of feedback can inform both users and exercise therapy experts about progress toward their therapy goals^76^. These types of feedback could thus enable robots to become standalone exercise coaches that help compensate for healthcare worker shortages^28^. Furthermore, formative feedback has been shown to have a positive impact on academic motivation and engagement^56,58^, test anxiety^56^, and attitude towards learning^56,77^. One can extrapolate that incorporating formative feedback into robot behavior has the potential to help robots gain long-term acceptance. Thus, the two types of feedback play different roles in facilitating user competence and should both be provided.

Summative feedback is currently the most common type of feedback provided by social exercise robots^23,24,78–80^. These robots utilize exergaming as their primary motivational strategy; such games provide summative performance feedback to try to change behavior^81^. The same sensors used to determine summative feedback can be leveraged by robots to provide formative feedback. When designing formative feedback, a one-size-fits-all approach is often unsuitable, as seen from the cue-based custom formative feedback we designed. Formative feedback uses acquired data to positively influence the individual’s learning if implemented correctly^70^. The feedback must be customized to the task at hand and must consider the user’s performance over the course of the task. Similarly, a robot could use adaptive formative feedback by changing its feedback strategies based on user performance over time^82^. One could additionally tune the difficulty of each task by adjusting the error threshold to match the user’s mental and physical capabilities, as done by Fitter et al.^24^ for older adult users. The error threshold for each task could change over time, starting large to make the task easy and reducing with subsequent success to elicit more precise performance of the desired action. Furthermore, drawing from dual-coding theory^83^, we recommend combining other interaction modalities with visual and gesture-based feedback. This idea is corroborated by our users, who recognized the different aspects of the feedback provided by our robot (head and arm gestures, facial expressions, and sounds) and wished it would also speak (Tables S5, S6).

One of this study’s strengths is the quantitative information gathered about user interactions with our robot. As motion-capture data is often extensive and hard to comprehend, we believe that heatmaps represent a practical alternative for exercise therapy experts seeking to observe and evaluate user performance. An interesting line of future research would be to validate the utility of individual heatmaps for use by exercise therapy experts. Furthermore, the Wasserstein distance metric could evaluate user performance over multiple sessions or days of exercise. The technical design of the Robot Interaction Studio^64^ enables us to provide consistent robot responses and interactions across all participants. Thus, the timing, type, and consistency of feedback were not affected by experimenter error or bias, as can happen with teleoperation. Finally, the use of educational assessments in such a context has not previously been explored in HRI. Though we examined them from the perspective of gesture-based interaction, these methods could generalize to any type of feedback mechanism.

One limitation of this study is the relatively small number of participants per condition, resulting in high diversity within each group; a larger participant pool could better elucidate how feedback types impact participant engagement and self-learning. When analyzing the results, we found that our participants could roughly be classified into two categories: explorers and imitators (Fig. S6). The explorers performed diverse actions, walking around the workspace, touching the robot, and actively trying to figure out the solution to each cue type. Conversely, the imitators stayed in a single position and mimicked the robot’s arm pose across all 75 trials, irrespective of any feedback provided. This imitation behavior could be linked to motor resonance^84^, which has been observed during HRI^85^, but it was the correct behavior for only one of the three cues. The imitators persisted with this behavior despite regular standardized reminders during breaks and when clarifications were requested. A questionnaire such as the Curiosity and Exploration Inventory^86^ might help explain participant strategy.

Exercise coach robots have the potential to improve compliance with exercise regimens in a range of settings including community rehabilitation. Ideally, such robots would guide users through exercise routines and provide effective feedback entirely autonomously. These robots would greatly benefit from providing compelling nonverbal feedback to their users, especially gestures, facial expressions, and sounds. However, effectively monitoring the user motion and adapting the robot’s behavior accordingly continues to be a technical challenge^21^ due to the speed and accuracy requirements for both perception and action. Furthermore, designing effective feedback response systems lies at the core of personalizing robotic systems to their users. As substantiated by the results of our study, feedback design based on formative and summative feedback holds great potential for future research in this area.

## Materials and methods

### Experiment platform

This study was designed to study nonverbal feedback from a social robot to a human user in a minimally supervised setting. Inspired by educational assessments, the feedback provided could be either formative or summative. We studied the effects of these two types of feedback using our Robot Interaction Studio^64^, which includes a Rethink Robotics Baxter Research Robot^65^ and ten cameras that are used for the markerless human-motion-capture system Captury Live^66^, plus three high-resolution cameras for human review of trials and facial expression analysis. The robot’s parallel-jaw grippers were equipped with foam boxing pads and sterile covers to make contact safe and comfortable.

### Cue and feedback design

The three task cues take inspiration from existing gesture-based HRI and the psychology literature, and the robot’s nonverbal feedback seeks to engage and challenge participants^54^. Prior work demonstrated that a robot needs to provide only minimal social cues to enhance the persuasiveness of the interaction^87^. Thus, we chose simple cues including sounds, emotions displayed on the robot’s face screen, and arm-based gestures to attract attention. Figure 1 shows the three cue types, their five variants, their associated types of formative feedback, and how summative feedback was conveyed across tasks. The cues and feedback are multimodal, blending the different types of nonverbal communication available to robots, which can be classified as gaze, gesture, mimicry, touch, movement, and interaction timing^3^. Utilizing multimodal feedback also aligns with our goal of leveraging education concepts such as dual-coding theory^62^.

Eye gaze is an integral element of human social interaction across all cultures^88^ and has thus been utilized by roboticists in many ways, such as indicating a robot’s mental state, directing user attention, and increasing fluidity in conversations^89^. Users have also been shown to prefer robots that move their heads^10^. Thus, we combined these ideas into a referential robot gaze directed at the user, as in Ref.^89^: Across all conditions of the study, the robot constantly looked at the user by turning its head to face them as they walked around the room. One high-resolution camera was mounted on top of the robot’s head to capture a view of the participant. The robot’s eyes periodically blinked to indicate awareness^24^, and the robot displayed verified open-source facial expressions^67^ and played open-source sound^90^ at key points in the interaction (Fig. 2A).

Each task cue was color coded: The location cue was represented by orange faces, the pose cue by purple faces, and the contact cue by gray faces. When presenting a task cue, the robot changed its face to an attentive expression (Figs. 6E, 7F, 8E). The robot maintained a neutral, non-smiling expression between cues. During cue presentation and while waiting for the participant to perform the task in all conditions, the robot exhibited attentiveness through a waiting expression that has pursed lips. When formative feedback was present (Fs and FS conditions), the robot smiled when the user performed the task correctly and had an attentive face otherwise. If a formative feedback sequence was triggered by the user’s error remaining above the threshold for 2 s, the face changed to a disappointed smirk while the formative gesture was being executed. After providing feedback, the robot’s expression returned to switching between the smile and the attentive face based on the error. A white face with a neutral expression appeared between task cues. The changes in the robot’s facial expression can be seen in Supplementary Video SV2.

All three cue types harness gesture-based interaction to convey meaning integral to the task. First, the location cue is a pointing (deictic) gesture that indicates a particular thing or area^3,91^ and is one of the first gestures learned by infants^92^. This type of gesture is well-explored within HRI^8^. The robot points to a location on the ground; without being told, the user needs to figure out they are supposed to walk to that location and stand there for the duration of the trial. We quantified the user’s error using the distance between the target and their current location on the floor, with a maximum radial error of 0.3 m considered correct. The robot provides formative feedback for the location cue by moving its arm to point at the feet of the user standing outside the target circle and then again at the target position; therefore, its arm motion shows the magnitude and direction of the user’s current task error.

Second, the pose cue invokes mimicry or imitation-based nonverbal communication^3^. Imitation is a fundamental technique children use to learn physical skills^93^ and develop social interaction abilities^94^, and pose imitation has been extensively used and validated in social robotics^23,78–80^. The tested variants of the pose cue are based on the Roboga Game by Fitter et al.^24^. Our robot held up both of its arms in a symmetric pose; without being instructed, the user had to figure out that they should mimic the robot’s arm pose for the duration of the trial. The user’s error for the pose cue was calculated by comparing the robot’s arms to the user’s arms after scaling and shifting them to have coincident shoulder joints; the error is the sum of the distances from each joint on the human arm to the closest segment on the respective robot arm with a maximum error of 0.15 m per arm considered correct. The formative feedback of the pose cue involves the robot moving its arms to an intermediate pose that is between the desired pose and the neutral pose, thereby demonstrating the amplitude of the user’s current error.

The third cue type was the contact cue; it highlights touch gestures, which have been shown to evoke positive feelings with users^3,95^. Similar to a high-five^96,97^, the robot held up one or both of its hands, palm(s) out. Again, without being told the goal, the user needed to figure out that they ought to walk into the robot’s workspace and touch its palm(s) with their hand(s) for the duration of the trial. For cues of this type, the user’s instantaneous error was calculated as the distance between the robot’s offered hand(s) and the closest human hand(s); it had a minimum threshold of 0.1 m for correct performance. For formative feedback with the contact cue, when the error is above this threshold, the robot wiggles its hand(s) an integer number of times equal to the error rounded up to the nearest meter.

Summative feedback was presented consistently across all cue types, involving one of three differently colored facial expressions with sounds that showed the quality of the user’s performance on the just-completed trial (Fig. 1C). If the participant error was less than the threshold for less than 10% of the 30-s trial time (< 3 s), the robot displayed a sad red face and played an unhappy sound. If the participant performed the task within the desired limits for any duration between 10 and 60% of the time (3–18 s), the robot displayed a slightly happy yellow face and played a matching sound. Finally, if the participant performed the task correctly for more than 60% of the trial (> 18 s), the robot displayed a happy green face and played a happy sound. All robot faces were adapted from previously validated facial expressions developed for the Baxter robot^67^. We carefully chose faces and sounds that were sufficiently distinct from each other and validated their interpretation through pilot testing. Additionally, Supplementary Video SV2 shows how our sample participant utilized these cues to his advantage. Supplementary Material Table S5 also shows that the majority of the participants noticed these cues.

### Study design

The study was designed as a mixed-design experiment with two between-subject factors with two levels each and one within-subject factor with three levels. The two between-subject factors were formative and summative feedback, with two levels each (with and without feedback). The within-subject factor was cue type, with the three levels being location cue, pose cue, and contact cue. We consider all five variants of each cue (different room locations, arm poses, and hand positions) to be largely equivalent to one another; thus, we do not consider the cue variant as a separate factor.

The study was approved by the Ethics Council of the Max Planck Society under the Haptic Intelligence Department’s framework agreement as protocol F007A. All methods were performed in accordance with the relevant ethics guidelines of the Max Planck Society and in accordance with the Declaration of Helsinki. All participants gave informed consent. Some participants also gave informed consent to the sharing of videos associated with their participation in the study; only sample trials from these participants are displayed in this paper. Participants were recruited from the Max Planck Institutes in Stuttgart, the University of Stuttgart, and a local Facebook group for foreigners. Every participant provided written informed consent. Participants who were not employed by the Max Planck Society received a compensation of 8€ per hour for participating in this study. The participant first filled out a Robot Evaluation Survey (Table S1), after which the experimenter read a set of pre-defined instructions out loud (Supplementary Materials Sect. 1). Participants often glanced at the experimenter or asked for further clarification; experimenters in typical studies help participants in such situations, but our experimenter provided no additional support other than repeating a shortened version of the instructions. Though frustrating, this policy was necessary to create a consistent experience of minimal supervision for all participants and allow us to investigate the effects of formative and summative feedback provided by a robot.

The participant then performed a total of 75 trials with 25 trials of each cue type. The repeating presentation order was as follows: a random location cue, followed by a random pose cue, and then a random contact cue. The flow of the interaction remained the same across all tasks and can be seen in Fig. 2A. At the beginning of a trial, the robot moved from the neutral pose to a cue-specific pose and then nodded its head to indicate that the trial was starting. Each trial lasted approximately 30 s after the robot moves into the desired pose. The system constantly checked whether the user was performing the desired task and calculated the score as the total duration of time that the task was performed correctly. If formative feedback was to be provided, it was triggered after 2 s in which the user error was continually greater than the defined threshold, and the provided feedback was based on the user’s error at the end of that interval.

To determine the presentation order, we first determined all possible combinations of one location cue, one pose cue, and one contact cue and then randomized the order of the combinations. To provide enough unique combinations for each group of participants, we created another randomized list of 125 combinations, resulting in a total of 250 combinations. The trial order for the first participant of each feedback group corresponded to combinations 1–25, the trial order for the second participant corresponded to combinations 26–50, and so on, such that the participants in each feedback group all experienced the same number and order of randomized trials. Participants were randomly assigned to one of the conditions; however, we took measures to balance the gender distribution. A brief break was provided to participants approximately after trials 25 and 50. After all 75 trials, the participant completed a cue evaluation survey to assess their understanding of each cue type, as well as a NASA Task Load Index (TLX) survey^98^ for each cue type. They also completed a post-study Robot Acceptance Survey and the System Usability Scale^99^ (Supplementary Materials Sect. 2).

### Participant information

The study included 28 participants with 7 participants in each group. We report the gender distribution as well as the age range, mean (M), and standard deviation (SD) for each group. The fs group (4 male, 3 female; aged 23–33, M = 28.71, SD = 3.35) received no formative or summative feedback. The Fs group (3 male, 4 female; aged 19–65, M = 31.43, SD = 15.45) received only formative feedback. The fS group (3 male, 4 female; aged 19–39, M = 29.00, SD = 6.14) received only summative feedback, and the FS group (4 male, 3 female; aged 22–42, M = 31.00, SD = 6.22) received both types of feedback. Due to consistent sentiments about robots across age groups^100^, we prioritized gender balance over age diversity in our study. The participants varied in their experience with robots, including 7 complete novices and 4 experts (Fig. S5A); only one participant had any substantial previous experience with Baxter (Fig. S5B). Most participants led relatively active lifestyles, reporting that they exercised several times a week (Fig. S5C), with some frequently attending exercise classes (Fig. S5D).

### Sources of data

Since the study was run within the Robot Interaction Studio, we had access to full-body markerless motion-capture data of the user, facial expression recognition from the camera on the robot’s head, and an additional Polar OH1^101^ optical heart-rate sensor worn around the user’s left bicep. For the purposes of this study, we narrowed these down to the following measures.

#### User error

User performance of each type of cue was determined by comparing the current value of the error metric defined for that task ($\epsilon (t)$) to a defined error threshold ($\bar{\epsilon }$), which was selected to provide a moderate level of difficulty for healthy adults. The precise formulations of the three error metrics are given by Eqs. (3), (4), and (5)/(6) in the Supplementary Materials Sect. 9.

#### User score

The value of the score (*S*) at time *t* for each cue type was calculated as a function of the error over time. Specifically, the score for a particular trial is equal to the total duration of time the user spent performing the task with an error less than the error threshold defined for that task. Scores varied continuously from 0 s to 30 s (perfect performance) and were calculated during the trial as follows, where $\Delta t$ is the time elapsed since the previous discrete time step:

1 $$\begin{aligned} S(t) = {\left\{ \begin{array}{ll} 0 &{} \text {if } t = 0\text { s} \\ S(t-\Delta t) + \Delta t &{} \text {if } \varepsilon (t) < \bar{\varepsilon } \\ S(t-\Delta t) &{} \text {otherwise}. \end{array}\right. } \end{aligned}$$

The mean $\Delta t$ was $0.03\mathrm {\,s}$, $0.16\mathrm {\,s}$ and $0.05\mathrm {\,s}$ for the location, pose and contact cue, respectively. The final score for a trial was taken to be the score at the trial’s end, i.e., $S(30\mathrm {\,s})$.

#### User grade

We created rubrics to evaluate the participants’ written post-study explanations of what the robot wanted them to do for each cue type. Participants received one point each for correctly identifying how the robot indicated the cue, their expected response, and the duration component of the task (Supplementary Materials Sect. 3). An external individual graded all explanations against these three-point rubrics with one-point resolution; they evaluated each set of responses in random order and were blinded to the participant’s feedback condition. The possible grades were 0, 1, 2, and 3 (perfect comprehension).

#### Distance traveled

This metric (*D*) can be defined as the total length of the path traversed by a participant over the 30 s duration of a trial. Let *k* be the distance traveled by the user across the floor in time step *t*. Then, this metric can be calculated as follows:

2 $$\begin{aligned} D = \sum _{0\,\text {s}}^{30\,\text {s}}k(t) dt. \end{aligned}$$

#### Time spent in the robot’s workspace

This metric is the percentage of trial time that a user’s hips were located within Baxter’s reachable workspace, which we defined as a circular region of the floor (radius $= 1.485$ m) centered on the robot’s torso, as seen in Fig. 5.

#### Attention

The videos from the camera placed on Baxter’s head were analyzed using the Affectiva AFFDEX^102^ facial-emotion-recognition algorithm integrated into iMotions^103^. It uses the participant’s head angle to determine how strongly they are attending to the robot (ranging from 0 to 100%), and we calculate the mean attention level per trial.

#### Heart rate

We collected participant heart rate throughout the study as beats per minute over time.

### Heatmap creation

Heatmaps were created by discretizing the relevant plane into a 20 $\times$ 20 grid and then binning the position data. Corresponding distributions representing the desired user behaviors for each cue type were created using a 2D normal distribution with a mean equal to zero and a standard deviation equal to the relevant error threshold divided by three. The spread of this distribution was chosen such that approximately 99.6% of the points fall within the desired area, to represent perfect user behavior. This distribution was then translated to be centered at the target location for the user’s hips or hand(s), depending on the cue type and variant. We generated the same number of points as the actual data. The Wasserstein metric was calculated between these desired distributions and the actual distributions.

### Statistical analysis

All data were analyzed in R (version 4.2.3) using non-parametric Aligned Rank Transform (ART) analysis of variance (ANOVA)^68^ due to the small sample size per group^104^ and because the data were non-normal. The unit of analysis for all analyses is the number of participants ($n=28$ where $n=7$ per group) except for the two-way ART ANOVA used for analyzing the Wasserstein metrics, which used the cue variants as the unit of analysis ($n=5$). Pairwise post-hoc comparisons were conducted using the multiple comparisons for the ART procedure^105^ with a Bonferroni correction and the Kenward-Roger method for approximating the degrees of freedom^106^. We report effect sizes using partial eta squared ($\eta _p$) for the ANOVA tests and Cohen’s *d* for t-tests^107^. The significance-testing level, $\alpha$, was considered to be 0.05 in all cases.

## Supplementary Information

Supplementary Information.

Supplementary Video 1.

Supplementary Video 2.

## Data Availability

The data associated with this manuscript can be accessed via 10.17617/3.UPWT1Q this link to Edmond, the research data repository of the Max Planck Society.
