# Supplementary material for: Closing the loop in minimally supervised human–robot interaction: formative and summative feedback
Source: Sci Rep. 2024 May 8;14:10564. doi: 10.1038/s41598-024-60905-x (PMC11079071; doi:10.1038/s41598-024-60905-x)
Supplement: Supplementary file 1 — Supplementary Information. [file 41598_2024_60905_MOESM1_ESM.pdf]

Supplementary Materials for

Closing the Loop in Minimally Supervised Human-Robot Interaction: Formative and Summative Feedback

Mayumi Mohan[1*], Cara M. Nunez[1,2], and Katherine J. Kuchenbecker[1*]

[1]Max Planck Institute for Intelligent Systems, Haptic Intelligence Department, Stuttgart, Germany
[2]Cornell University, Sibley School of Mechanical and Aerospace Engineering, USA
[*]Corresponding author emails: maymohan@is.mpg.de, kjk@is.mpg.de

Supplementary Text
Tables S1 to S6
Figures S1 to S16
Legends for Videos SV1 and SV2
References *(103–110)*

Other supplementary materials for this manuscript:
Videos SV1 and SV2

1 Experimenter Script

The experimenter read the following script to the participant at the start of the study:

> This study is designed to understand how humans react to different robot behaviors during a movement-focused activity session with a humanoid robot. In each trial of the study, the robot will present you with a command communicated using movements of the robot's arms and head, facial expressions on the robot's screen, and sounds. You will have a set amount of time to complete the desired task communicated by the robot. Once the time has run out, you will move on to the next trial, and the robot will present a new command.
>
> We ask that you please stay in front of the marked line (*experimenter pointed at tape line on the floor*) and do not stick your hand or arm into the joints of the robot (*experimenter pointed out Baxter's joints*). You can ignore the four bits of tape on the floor. In order to complete the desired task, you may need to move throughout the room and make contact with the robot.
>
> We will be collecting both video and audio recordings, as well as heart rate data. Once you have put the heart rate sensor on your left upper arm, we ask that you move like this (*experimenter showed arm movements desired for Captury Live calibration*) so that we can confirm that the necessary data is being collected. The trials are broken into three blocks, and you will get a break between blocks. After each of the breaks, we will ask you to perform these similar movements to confirm that the necessary data is still being collected.
>
> After you have completed all three blocks of the study, you will complete a set of surveys about your experiences interacting with the robot.
>
> If at any point you would like to end the study, please let me know. What questions do you have?
>
> If there are no further questions, we can get started. Please do your best to interpret the robot's cue and complete the desired task.

Beyond this basic introduction, participants were not given any explicit instructions about what to do in the study. After each break, the experimenter read the following text:

> As a reminder, stay in front of the marked line. You can ignore the four bits of tape on the floor. Please do not touch the joints of the robot; otherwise, you may move around (*experimenter gestured arm broadly to indicate the wide workspace*) and make contact with the robot to complete the task. Please do your best to interpret the robot's cue and complete the desired task.

If the participant asked any further questions or requested assistance with the tasks, the experiment repeated the following statement:

> Please do your best to interpret the robot's cue and complete the desired task.

2 Administered Surveys

The following surveys were administered to each participant via the secure online platform LimeSurvey.

Table S1. Robot Acceptance Survey (RAS)[109].

I think using the robot is a **good** idea.
I am afraid to make **mistakes** while using the robot.
I am afraid to **break** something while using the robot.
People would be **impressed** if I had such a robot.
Robots are **nice** to work with.
I am afraid that I could lose my **job** because of a robot.
I could **cooperate** with the robot.
I think the robot is **easy** to use.
I **like** the presence of the robot.
I feel **threatened** by the robot.
This robot would be **useful** to me.
This robot could **help** me.
This robot could **support** me.
I consider the robot to be a **social** agent.
I feel **understood** by the robot.
I feel **comfortable** while interacting with the robot.
I could work with the robot, **if** someone **helped** me.
I could work with the robot **without** any **help**.
I could work with the robot, if I had good initial **training**.
I **trust** the robot.
I would **follow** the example of the robot.

At the beginning of the study:

Please rate how much you agree or disagree (continuous slider bar, strongly disagree – strongly agree) with each of the following statements (shown in Table S1).

This Robot Acceptance Survey (RAS) was developed by Weiss et al.[109].

After completing the study:

1. Please rate how much you agree or disagree (continuous slider bar, strongly disagree – strongly agree) with each of the following statements (shown in Table S2).

   This System Usability Scale (SUS) was developed by Brooke[100].

Table S2. System Usability Scale (SUS)[100].

I think that I would like to use this system frequently.
I found this system unnecessarily complex.
I thought this system was easy to use.
I think that I would need assistance to be able to use this system.
I found various functions in this system were well integrated.
I thought there was too much inconsistency in this system.
I would imagine that most people would learn to use this system very quickly.
I found this system to be very cumbersome/awkward to use.
I felt very confident using this system.
I needed to learn a lot of things before I could get going with this system.

2. Please rate how much you agree or disagree (continuous slider bar, strongly disagree – strongly agree) with each of the following statements (shown in Table S3).

   This NASA-TLX was developed by Hart and Lowell[99].

3. Cue evaluation (Table S4) for the Location cue

Table S3. NASA Task Load Index (TLX)[99].

How mentally demanding was the task? (continuous slider bar, Very Low – Very High)
How physically demanding was this task? (continuous slider bar, Very Low – Very High)
How hurried or rushed was the pace of this task? (continuous slider bar, Very Low – Very High)
How successful were you in accomplishing what you were asked to do? (continuous slider bar, Failure – Perfect)
How hard did you have to work to accomplish your level of performance? (continuous slider bar, Very Low – Very High)
How insecure, discouraged, irritated, stressed and annoyed were you? (continuous slider bar, Very Low – Very High)

4. Cue evaluation (Table S4) for the Pose cue
5. Cue evaluation (Table S4) for the Contact cue

Table S4. Cue Evaluation

In your own words, please describe what the robot is telling you to do in this video (Separate videos were shown for location cue, pose cue and contact cue. Versions of these videos can be seen in Supplementary Video SV1. The videos shown to the participants were somewhat zoomed in and silent).
How confident do you feel about your understanding of this robot cue? (continuous slider bar, Not Very Confident – Very Confident)
For the trials when the robot gave you this type of cue, respond to the questions in Table S3.
For cues of this type, how often did you perform (or not perform) movements only to find out how the robot would react? (multiple choice: never, only in one trial, in a couple trials, in several trials, in many or all trials)
Please explain. (optional open response)
Do you have any other comments about this type of cue? (optional open response)

6. Feedback evaluation
   (a) What feedback did you receive from the robot? (check all that apply: head motions, arm gestures, facial expressions, lights on the hands, nonverbal sounds, speech, other (with text entry))
   (b) In your ideal scenario, what feedback do you wish the robot would provide? (check all that apply: head motions, arm gestures, facial expressions, lights on the hands, nonverbal sounds, speech, other (with text entry))
7. Please rate how much you agree or disagree (continuous slider bar, strongly disagree – strongly agree) with each of the following statements (shown in Table S1).
8. Do you have any additional comments about the system or the study? (optional open response)
9. Demographics questionnaire
   (a) Age (open response)
   (b) Gender (multiple choice: male, female, other)

(c) Profession (open response)

(d) Is your education/background technical? (multiple choice: yes, no)

(e) Please rate your level of experience interacting with robots (prior to this study): (multiple choice:
Complete novice – I have never used a robot before today
Beginner – I have played with commercial robot toys
Intermediate – I have interacted with more sophisticated robots (like Pepper, NAO, Baxter, Kuka arm, Kinova arm, etc.)
Proficient – I have some experience interacting with and designing, building, and/or programming different kinds of robots
Expert – I frequently design, build, program, and/or work with robots on my own)

(f) Please rate your level of experience interacting with a Baxter robot (prior to this study): (multiple choice:
Complete novice – I did not know what a Baxter robot is before today
Beginner – I have seen and/or read about a Baxter robot before
Intermediate – I have interacted with a Baxter robot via robot demos or other activities
Proficient – I have some experience interacting with and programming a Baxter robot
Expert – I frequently program and/or work with a Baxter robot)

(g) How often do you exercise? (multiple choice:
Never
Once a month
Every other week
Once a week
Several times a week)

(h) How many times have you taken fitness or dance courses like yoga, Zumba, gymnastics, or ballet? (multiple choice:
Never
One to three times
Between four and ten times
Between eleven and one hundred times
More than one hundred times)

(i) Hometown (where you grew up): city and country (open response)

3 Participant Response Grading Rubrics

The following rubrics were provided to the external, impartial grader to evaluate participant responses for their understanding of each of the cue types (the first question in Table S4). The grader was asked to focus on content, not specific words, and was allowed to award only integer scores, with no partial points. Thus, the possible scores were 0, 1, 2, or 3 points, with 3 representing perfect comprehension of the robot cue in question.

For grading responses pertaining to the location cue, the total score for their response is the sum of the points earned for describing each of the following:

1 Point	Robot is pointing to location/position/place
1 Point	Participant must move to location/position/place/through the workspace
1 Point	Participant must stay in location/position/place

For grading responses pertaining to the pose cue, the total score for their response is the sum of the points earned for describing each of the following:

1 Point	Robot is making a pose
1 Point	Participant must mimic/copy pose
1 Point	Participant must hold pose

For grading responses pertaining to the contact cue, the total score for their response is the sum of the points earned for describing each of the following:

1 Point	Robot is lifting hand/holding out hand for contact
1 Point	Participant must make contact with robot's hand pads
1 Point	Participant must make repeated contact or hold contact with robot's hand pads

4 Participant Survey Responses

We administered surveys to the participants as outlined in Section 2. Here we present the responses to these surveys.

NASA-TLX Results

Fig. S1 shows the results of the NASA Task Load Index, with one data point for each participant's experience of each cue. As workload is a subjective indicator, high variations among participants are expected. Visual inspection hints that the location cue may have had higher workload than the other cues, and that summative feedback might have slightly increased workload for this cue alone. Visual inspection additionally shows that both kinds of feedback may have reduced participants' overall workload for the pose cue and contact cue. However, a three-way mixed ART ANOVA showed that there was a significant three-way interaction between formative feedback, summative feedback and cue type on the total TLX results ($F(2,48) = 3.4, p = 0.04, \eta_p^2 = 0.13$). However, post-hoc analyses did not show any significant effects, likely due to the relatively low number of participants for this highly scattered between-subjects measure.

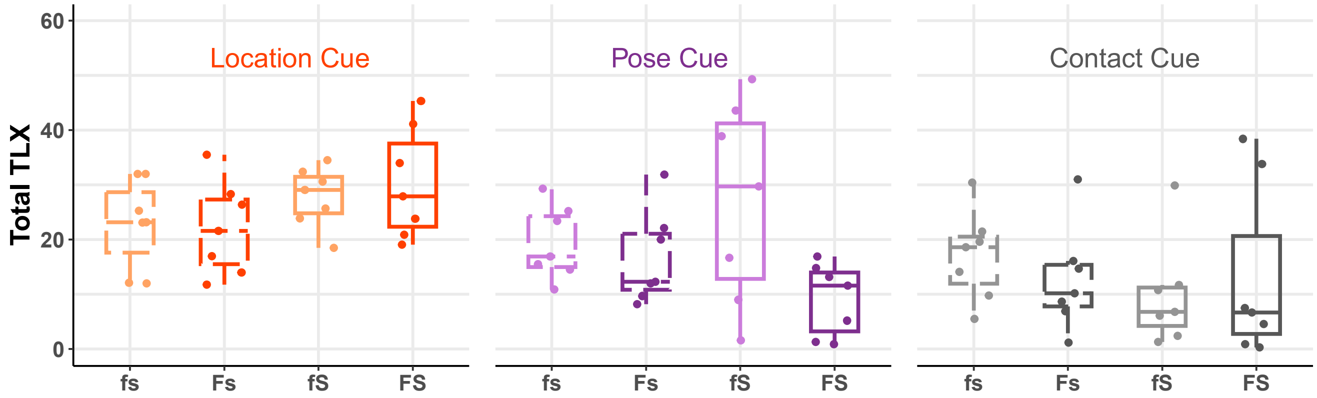

Figure S1. Self-reported total NASA-TLX workload results across the three cue types and the four feedback conditions (fs, Fs, fS, and FS). Table S3 shows the six questions that constitute this survey; higher values indicate greater workload. Though there was a significant three-way interaction, post-hoc results were not significant.

SUS Results

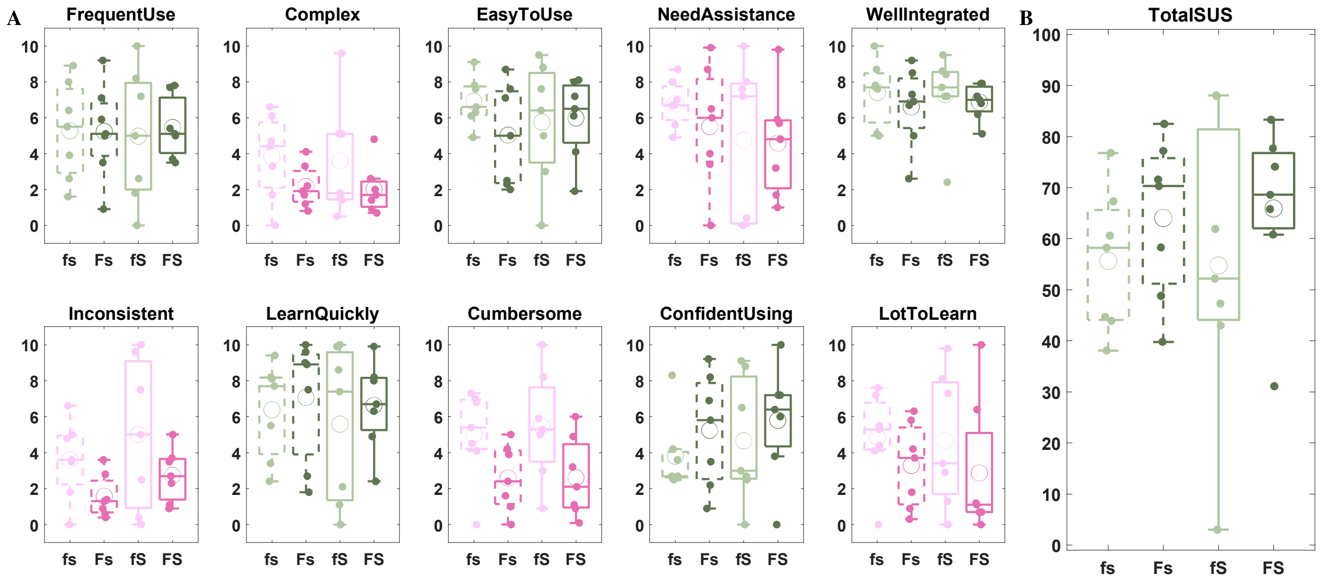

Figure S2. System Usability Scale results across the four feedback conditions (fs, Fs, fS, and FS). **(A)** Plots of the responses to the ten individual questions that make up this survey (Table S2). Positive questions are colored green, and negative questions are colored pink. **(B)** Total score. Responses to negative questions were inverted before summing with the positive responses to yield the total score shown.

Fig. S2 presents the results of the System Usability Scale, which we analyzed by question and overall. Again, variability between participants is high for this subjective measure. Overall, a two-way ART ANOVA revealed that feedback condition had a significant impact on only one item. There was a main effect of formative feedback on how cumbersome the usability of the system was $(F(1,24) = 7.81, p = 0.01, \eta_p^2 = 0.25)$. Participants found that formative feedback made the system significantly less cumbersome to use $(t(24) = 2.79, p = 0.01, d = 1.06)$. There was also a statistically trending effect of formative feedback on how inconsistent users from the system to be $(F(1,24) = 3.89, p = 0.06, \eta_p^2 = 0.14)$. As seen in Fig. S2A, formative feedback may have made the system seem more consistent. Statistical analysis indicated that feedback condition did not significant affect the total SUS ratings. However, visual inspection shows that formative feedback may tend to elicit higher system usability scores (mean $\pm$ standard deviation for fs: 55.7 ± 14.0, Fs: 64.1 ± 15.6, and FS: 65.9 ± 17.1), likely stemming from the two questions (cumbersome, inconsistent) with significant or trending benefits of formative feedback. Summative feedback does not appear to have affected this measure (fS: 54.8 ± 29.3). In retrospect, the System Usability Scale was not an ideal qualitative measure to use in this study, since it did not focus on the feedback itself.

RAS Results

Figs. S3 and S4 show the results of the Robot Acceptance Survey, which was administered both before and after the study. Overall, participants had a more negative attitude towards robots after the study. This view could be a result of the frustration they felt due to the lack of instructions. The RAS results were split into eight categories as recommended by Weiss et al.[109]. Fig. S3 shows the results of four of these categories (performance expectancy, effort expectancy, attitude towards technology and self-efficacy) which were adapted from the UTAUT model[110]. Fig. S4 depicts the other four categories (forms of grouping, attachment, reciprocity, cultural context), which were derived from the concept of object-centered sociality[111]. Each component of the RAS was analyzed using a three-way ART ANOVA that used the two feedback types (without and with formative feedback, without and with summative feedback) and study participation (before and after the study) as the three independent factors.

Fig. S3A shows the three RAS items that measure participants' expectations of robot performance. The study had an impact on how users felt about the usefulness $(F(1,24) = 12, p = 0.0018, \eta_p^2 = 0.34)$, helpfulness $(F(1,24) = 8, p = 0.0094, \eta_p^2 = 0.25)$ and supportiveness of the robot $(F(1,24) = 7.3, p = 0.012, \eta_p^2 = 0.23)$. Fig. S3A shows that participants felt that robots are less useful $(t(24) = 3.5, p = 0.0018, d = 0.94)$, less helpful $(t(24) = 2.8, p = 0.0094, d = 0.75)$, and less supportive $(t(24) = 2.7, p = 0.012, d = 0.72)$ after the study.

Fig. S3C shows participants' expectations of the effort required to use the robot. Although there was no effect of feedback or study participation on the perceived ease of use of the robot (Fig. S3C), the study significantly affected how users felt about cooperating with the robot $(F(1,24) = 4.3, p = 0.05, \eta_p^2 = 0.15)$. They felt that they could cooperate less with the robot after the study $(t(24) = 2.1, p = 0.05, d = 0.55)$. This response could be attributed to the lack of instructions and participants' reduced feelings of self-efficacy.

Participants' attitudes toward technology were measured via three questions (Fig. S3B). The study had a significant impact on whether they thought that using the robot is a good idea $(F(1,24) = 14, p = 0.00099, \eta_p^2 = 0.37)$. After the study, they had a lower opinion about using the robot $(t(24) = 3.7, p = 0.00099, d = 1)$. There was a significant interaction between formative feedback and participation in the study on how afraid they were to make mistakes $(F(1,24) = 4.3, p = 0.049, \eta_p^2 = 0.15)$; visual inspection of Fig. S3B indicates that participants who received formative feedback (Fs, FS) may have been less afraid of making mistakes after the study, but we did not find any significant simple effects upon further analyzing this two-way interaction. The study also had a significant impact on the user's feeling that they may break the robot $(F(1,24) = 9, p = 0.0061, \eta_p^2 = 0.27)$; they felt that they were substantially less likely to break it after the study $(t(24) = 3, p = 0.0061, d = 0.8)$.

The final category of the UTAUT-based questions measured self-efficacy (Fig. S3D). Perceived self-inefficacy increases arousal and anxiety and makes people feel they cannot perform[112]. On the other hand, a positive sense of self-efficacy reduces fear and increases the person's ability to perform 'threatening' tasks[113] and is associated with acceptance for all robots[114]. There was no impact of either feedback or the study on feelings of comfort in the presence of the robot. The presence of summative feedback had a significant impact on how users felt about whether they could use the robot with external help $(F(1,24) = 5.1, p = 0.033, \eta_p^2 = 0.18)$. The presence of summative feedback made users feel that they could work with the robot if help was provided $(t(24) = 2.3, p = 0.033, d = 0.66)$. There was a statistically significant two-way interaction between formative feedback and the study on being able to work with the robot without external help $(F(1,24) = 4.3, p = 0.049, \eta_p^2 = 0.15)$. Though we did not find any significant simple effects upon further analyzing the two-way interaction, visual inspection appears to indicate that the presence of formative feedback made participants feel like they could use the robot without help. There was a significant three-way interaction between formative feedback, summative feedback, and participating in the study on being able to use this system with initial training $(F(1,24) = 7.5, p = 0.012, \eta_p^2 = 0.24)$. Post-hoc analyses revealed that there was a two-way interaction between both types of feedback after the study $(F(1,24) = 10, p = 0.0079, \eta_p^2 = 0.3)$. Additionally, the presence of summative feedback had a significant impact when formative feedback was absent $(F(1,12) = 7.6, p = 0.035, \eta_p^2 = 0.39)$

but not when formative feedback was present. In this case, participants thought it would harder to work with the robot when training was provided when summative feedback was absent ($t(12) = 2.8, p = 0.017, d = 1.5$). Overall, participants felt they would require some form of assistance or initial training to be able to use the system. This observation can be attributed to the fact that instructions were not provided to the participants to allow the effects of the autonomous robot's feedback to be measured. Future research could investigate the impact of feedback on self-efficacy in more detail.

Fig. S4 looks at user attributions of social and cultural constructs toward robots. There were no effects of the study or feedback conditions on participants' desire to build groups with and around the robot (Fig. S4A), their feelings of attachment toward robots (Fig. S4B), or their feelings of reciprocity (Fig. S4C). Finally, Fig. S4D looks at participant robot perceptions from a cultural context. The study had a negative effect on participant perceptions of impressing others with robot ownership ($F(1,24) = 6.1, p = 0.021, \eta_p^2 = 0.2$); users felt it would be less impressive to own a robot after the study ($t(24) = 2.5, p = 0.021, d = 0.66$). The study also had an impact on fear of losing one's job because of a robot ($F(1,24) = 8.5, p = 0.0076, \eta_p^2 = 0.26$); participating in the study made them less afraid of losing their jobs due to a robot ($t(24) = 2.9, p = 0.0076, d = 0.78$). However, their feelings about how nice robots are did not change after the study.

Robot Behavior Results

To understand the influence of combining robotic arm gestures, head motions, facial expressions, and sounds to convey the task cues and associated feedback, we asked participants to identify the ways in which the robot had communicated. Table S5 shows which robot actions participants remembered experiencing during the study. All but one of the participants noticed the head movements of the robot; the face screen rotated to face the user throughout the study in all feedback conditions. Interestingly, four of the 28 participants (14.3%) did not indicate that they saw the robot make arm gestures even though it moved its arms to convey every cue in the study. All participants who had summative feedback (fS, FS) noticed that the robot had facial expressions, and all but one of them noticed the robot's nonverbal sounds. Most participants without summative feedback also noticed the facial expressions and nonverbal sounds. All participants correctly identified that the robot did not have lights on its hands or speak. In summary, participants across all feedback conditions correctly noticed the multimodal aspects of the robot's behavior.

Table S6 shows the robot actions participants wished for. Interestingly, most participants (85.7%) wished for the robot to speak, a modality commonly used by robots but not employed in this study to focus on nonverbal communication. Facial expressions (78.6%), head gestures (67.9%), and arm gestures (60.7%) were also requested by more than 60% of participants. Only about half (53.6%) of users wished for nonverbal sounds. A minority (28.6%) wished for lights on the robot's hands, though none of the participants who received both formative and summative feedback (FS) were interested in such a feature. Interestingly, the participants in the formative group had the lowest number of individuals expressing a desire for speech. These preferences validate the important role non-verbal communication can play in complementing verbal cues.

Table S5. Number of participants who identified each listed type of action as being provided by the robot during the study, separated by feedback condition.

	fs	Fs	fS	FS	Total %
**head gestures**	7	7	6	7	96.4%
**arm gestures**	6	6	5	7	85.7%
**facial expressions**	5	6	7	7	89.3%
**lights on the hands**	0	0	0	0	0.0%
**nonverbal sounds**	5	4	7	6	78.6%
**speech**	0	0	0	0	0.0%

Table S6. Number of participants who wished for each listed type of action from the robot, separated by feedback condition.

	fs	Fs	fS	FS	Total %
**head gestures**	3	6	6	4	67.9%
**arm gestures**	3	5	5	4	60.7%
**facial expressions**	5	6	7	4	78.6%
**lights on the hands**	3	2	3	0	28.6%
**nonverbal sounds**	3	3	6	3	53.6%
**speech**	7	5	6	6	85.7%

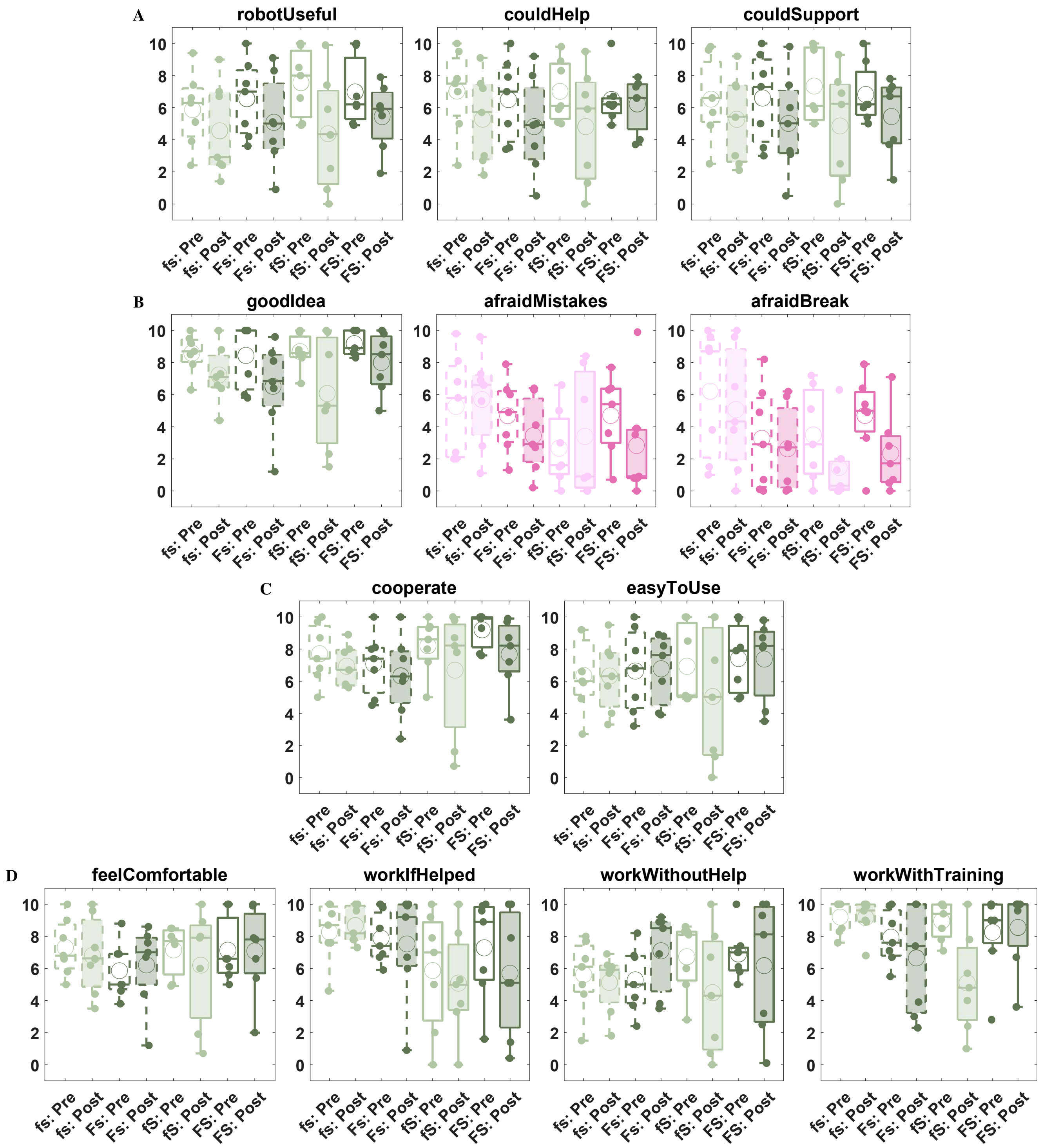

Figure S3. Robot Acceptance Survey categories based on the UTAUT model across the four feedback conditions (fs, Fs, fS, and FS). Positive questions are colored green, and negative questions are colored pink. **(A)** Participant responses regarding performance expectancy of robot. They felt that the robot was less useful, less helpful, and less supportive after the study. **(B)** Participant responses to questions assessing their attitudes toward technology. Overall, after the study they were less confident that using that robot was a good idea and were less afraid to break the robot after the study. **(C)** Participant responses about effort expectancy. They felt less able to cooperate with the robot after the study. However, the study had no impact on ease of use. **(D)** Participant responses to self-efficacy measures. Summative feedback made participants feel like they could work with the robot if external help was provided. There is also some indication that the presence of formative feedback made them think they could work with the robot without help.

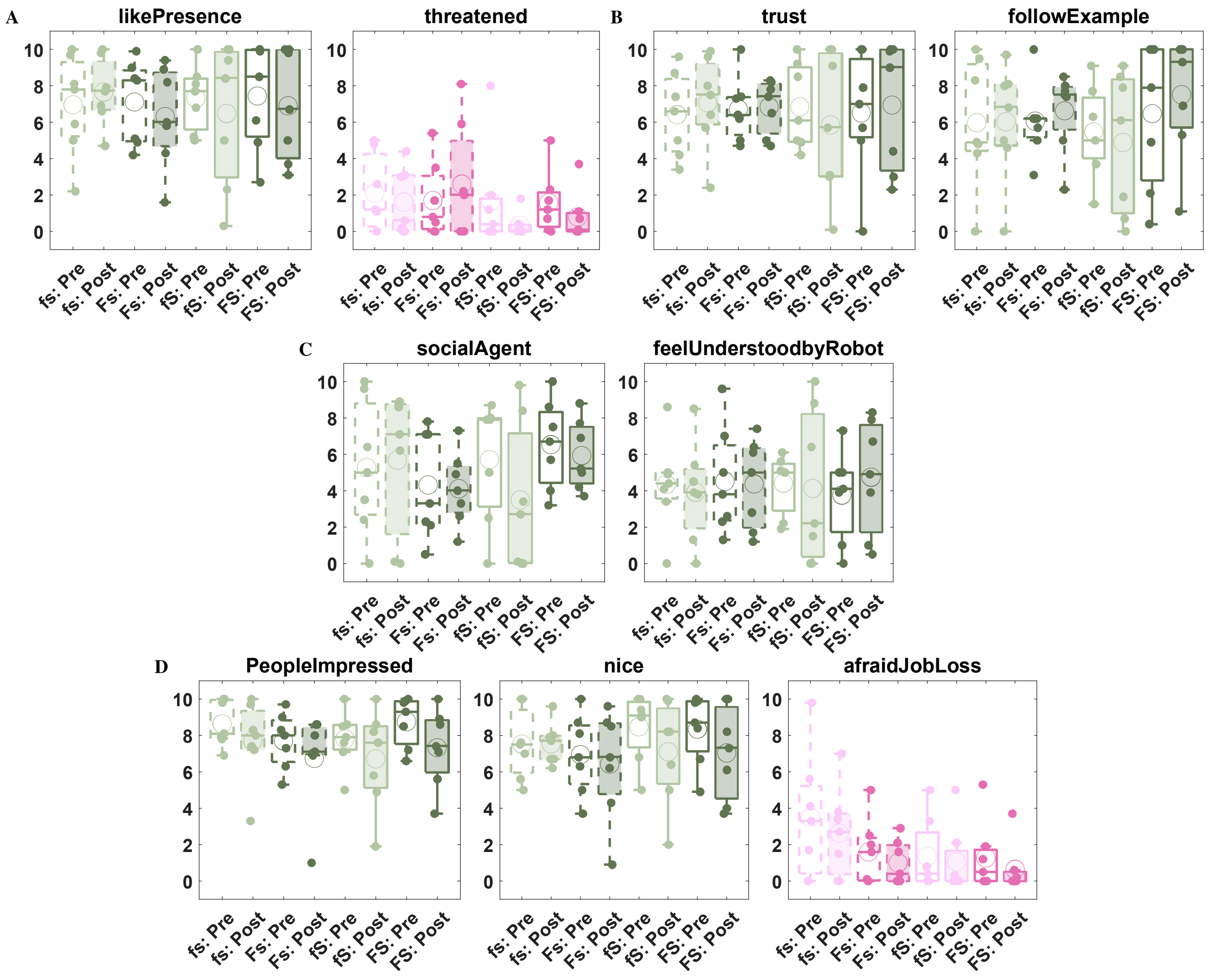

Figure S4. Robot Acceptance Survey categories based on the concept of object-centered sociality across the four feedback conditions (fs, Fs, fS, and FS). Positive questions are colored green, and negative questions are colored pink. **(A)** Participant responses to forms of grouping category items. There are no effects of the feedback types or the study on either liking the presence of the robot or feeling threatened. **(B)** Participant responses regarding attachment toward robots. There are no effects of the study or feedback on feelings of trust or the user's desire to follow the example of the robot. **(C)** Feelings of reciprocity reported by participants. There are no effects of the study or feedback on feelings of social agency of the robot or being understood by the robot. **(D)** Participant responses to cultural perception of robots. After finishing the study, participants felt that others would be less impressed if they owned a robot. They also were less afraid that they might lose their job because of a robot.

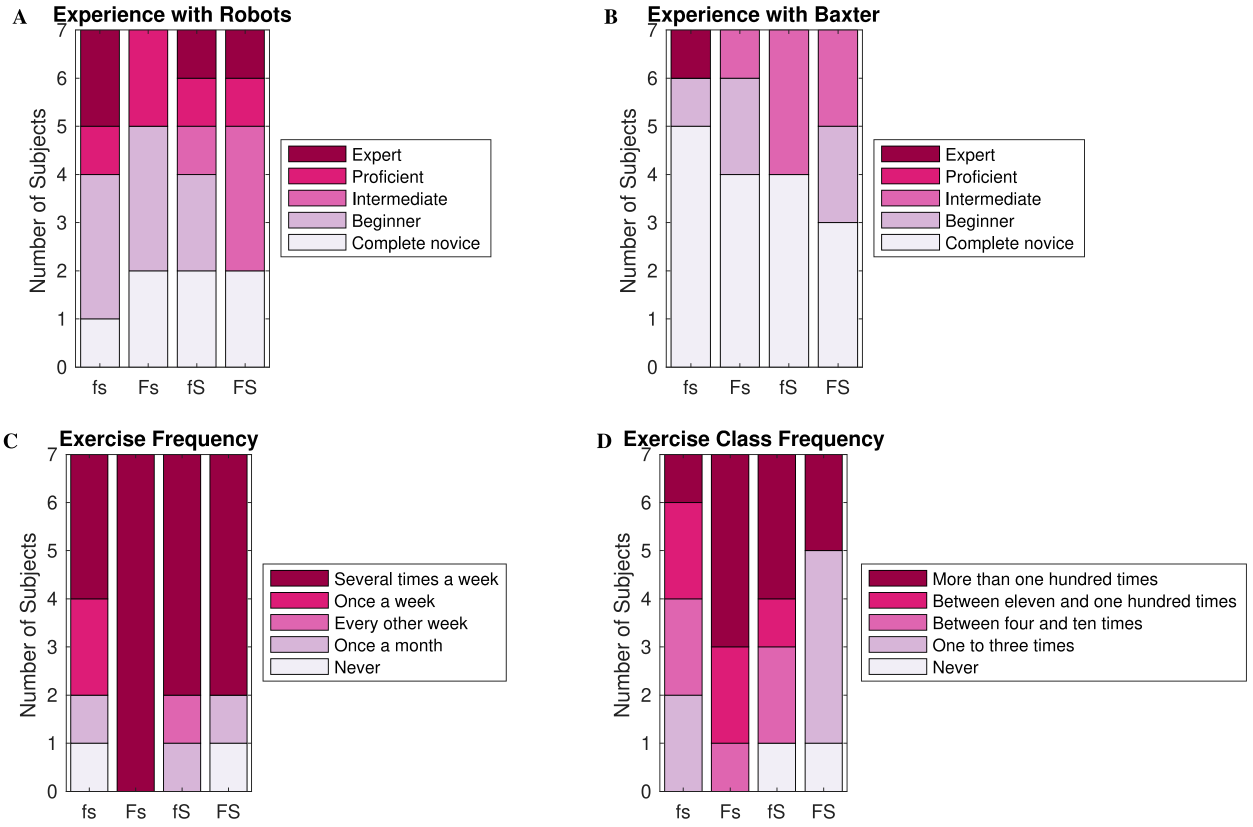

Figure S5. **(A)** Participant self-reported experience with robots. **(B)** Participant self-reported experience with a Baxter robot. **(C)** Participant self-reported exercise frequency. **(D)** Participant self-reported experience with exercise classes.

Participant Experience with Robots and Exercise

Fig. S5 shows how participants responded to four questions about their prior experience with robots, experience with Baxter, exercise frequency, and exercise class frequency. The full text of these questions is listed in Sec. 2

Participant Self-Reported Cue Exploration Measures

The post-study survey asked participants to report how often they performed movements only to find out how the robot would react, i.e., to explore how the system worked. We found that there was a significant effect of cue type on this metric ($F(2,48) = 7.4, p = 0.0015, \eta_p^2 = 0.24$). Participants explored robot reactions much more for the location cue in comparison to the pose cue ($t(48) = 3.4, p = 0.0042, d = 0.91$) and the contact cue ($t(48) = 3.3, p = 0.0058, d = 0.88$). This finding matches well with the lower scores and lower comprehension that participants achieved in the location cue, as exploring how the robot reacts is a good way to improve one's comprehension and thus performance.

While analyzing these responses and the corresponding comments about trying different actions to explore robot behavior, we noticed that this question was interpreted in two distinct ways. Some participants assumed that this question was asking about actions they performed to elicit the right response from the robot. This was not our intended meaning, as we expected all participants to try to figure out the cues; this ambiguity probably reduced the power of this question. Others interpreted this question as we intended, asking about erroneous actions they intentionally performed to generate robot feedback after identifying the correct way to respond to a particular cue. Participants who answered positively with this interpretation reported to have enjoyed testing robot actions to see the formative feedback, several participants with formative feedback made similar anecdotal comments to the experimenter.

Participant Confidence in Cue Comprehension

The post-study survey also asked participants to rate their confidence in their explanation of the meaning of each of the robot's cues. The comprehension scores appear in Fig. 4, and the corresponding confidence results are shown in Fig. S7, separated by feedback condition. Participants used the entire range of confidence values from 0 to 10, as this self-reported measure is highly subjective. Confidence was generally highest for the contact cue, followed by the pose cue, and then the location cue. A three-way ART ANOVA showed there was a statistically significant two-way interaction between formative feedback and

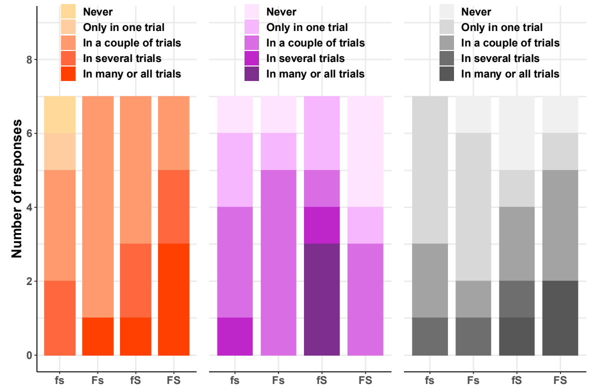

Figure S6. Participant self-reported frequency of exploring how the robot would react to different movements.

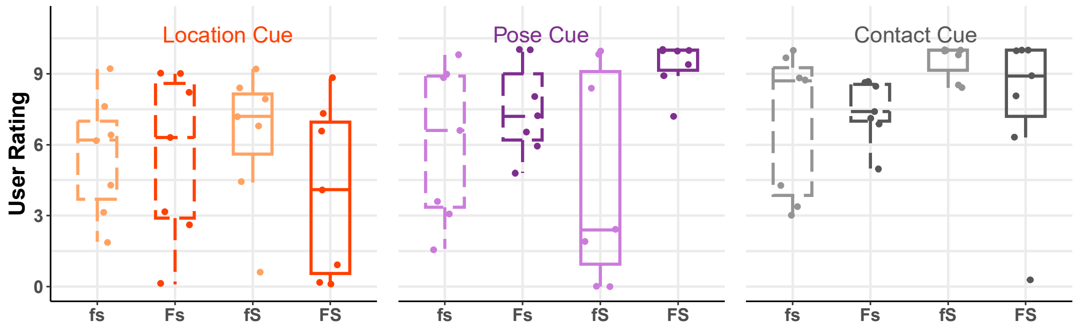

Figure S7. Participant confidence in their explanation of each of the robot's cue types.

cue type on the user's confidence (Fig. S7) at interpreting the cue $(F(2,48) = 4.1, p = 0.023, \eta_p^2 = 0.15)$. However, we did not find any significant effects upon conducting post-hoc analyses. Visual inspection seems to show that the combination of formative and summative feedback (FS) might have made participants most confident of their understanding of the pose cue, and only summative feedback (fS) may have elicited the highest confidence for the location cue and contact cue. Interestingly, formative feedback points out mistakes as the user makes them and might have thus made participants more aware of their errors, reducing their confidence while also increasing their comprehension; the relationship between feedback, task difficulty, task comprehension, and confidence in comprehension deserves further study.

5 Participant Heart Rate

Fig. S8 shows the average participant heart rate separated for the four feedback conditions; we see a wide range of values from below 60 bpm to above 130 bpm due to individual physiological differences. Formative feedback has been qualitatively shown to reduce test anxiety[56]. Thus, a proper measurement and analysis of heart-rate data could have the potential to test this observation. In the future, we recommend collecting a baseline resting heart rate so that such measurements can be properly normalized by participant and then analyzed.

6 Missing Data

Our validation of the Robot Interaction Studio[64] involved the three cues tested in this study and no feedback related to the task. Thus, we utilized the data from this prior work as our baseline condition, without formative or summative feedback (fs). The initial analysis of the score and error data from the fs condition was missing 28.36% of the interactions (from 0 to 23 trials per participant for a total of 116 missing trials), as described by Mohan et al.[64], due to a bug in the data-logging software. Since these participants did not experience any feedback, we were able to successfully recover all of the lost data by reprocessing the raw motion-capture data, which had been saved separately. Thus, our reported data set is complete for the fs condition. However, we do not have any data for two trials for one participant in the Fs condition due to an experimenter error. For the purposes of statistical analysis, these two trials were populated with the median of the dependent variable in question based on the other trials of the same cue type recorded for this participant.

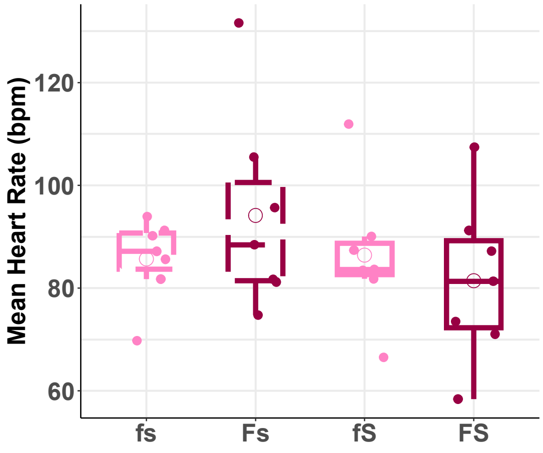

Figure S8. Mean participant heart rate during the study separated by feedback conditions. This indicator was measured in beats per minute (bpm).

7 Participant Activity Metrics

Fig. S9 depicts the three participant activity measures that we looked at. Fig. S9A shows the average total distance participants traveled during each trial. Figs. S9B and S9C depict the mean attention level the user paid to the robot and the significant effects of the three cue types on this metric. Finally, Figs. S9D and S9E show the mean time users spent within the workspace of the robot and the significant effect that the presence of summative feedback had on this metric.

8 Complete Heatmaps for All Conditions

This section presents all the heatmaps generated across all three cue types and feedback types; see Figs. S10, S11, and S12. Unlike the universal color scaling used in the main manuscript, the color intensities in these heatmaps have been scaled to the maximum value per cue type per feedback for better visibility of user actions.

9 Error Metric Definitions

We mathematically defined the error metric for each type of cue as a function of the desired body location or pose associated with the task variant and the motion-capture data presently measured for the participant. All three error metrics are linear distances measured in meters.

The error metric for the location cue (ε_ℓ) at time t was defined as the 2D Euclidean distance between the desired position ($\vec{p}$) where the robot was pointing to and the current projection of the user's hip position onto the ground ($\vec{u}_g(t)$), as follows:

$$\varepsilon_\ell(t) = \left|\vec{p} - \vec{u}_g(t)\right| \tag{3}$$

The associated threshold was $\bar{\varepsilon_\ell} = 0.3\,\text{m}$.

The error metric for the pose cue (ε_p) at time t is a curve-similarity measure based on Frechet's distance[115]. For each robot arm, this metric can be defined as the sum of the shortest distance from every joint on the robot arm to a line-segment-based representation of the corresponding human arm. This metric is calculated after performing homogeneous transformations to scale the human arms to the length of the robot arms, mirror them, and transform them to start at the same locations as the robot arms. Each robot arm has $n = 7$ revolute joints. We define $d_{Lj}(t)$ to be the shortest distance from the current location of the reference frame attached to joint j of the left robot arm to the closest segment on the corresponding human arm; $d_{Rj}(t)$ has the same meaning for the robot's right arm. To ensure that both human arms are mimicking the pose of the respective robot arm, we define the overall pose error to be the maximum of the errors for the left and right arms, as follows:

$$\varepsilon_p(t) = \max\left(\sum_{j=1}^{n} d_{Lj}(t), \sum_{j=1}^{n} d_{Rj}(t)\right) \tag{4}$$

Thus, the error metric for the pose cue had to fall below the error threshold ($\bar{\varepsilon_p} = 0.15\,\text{m}$) for both arms simultaneously for the user's action to be judged as correct.

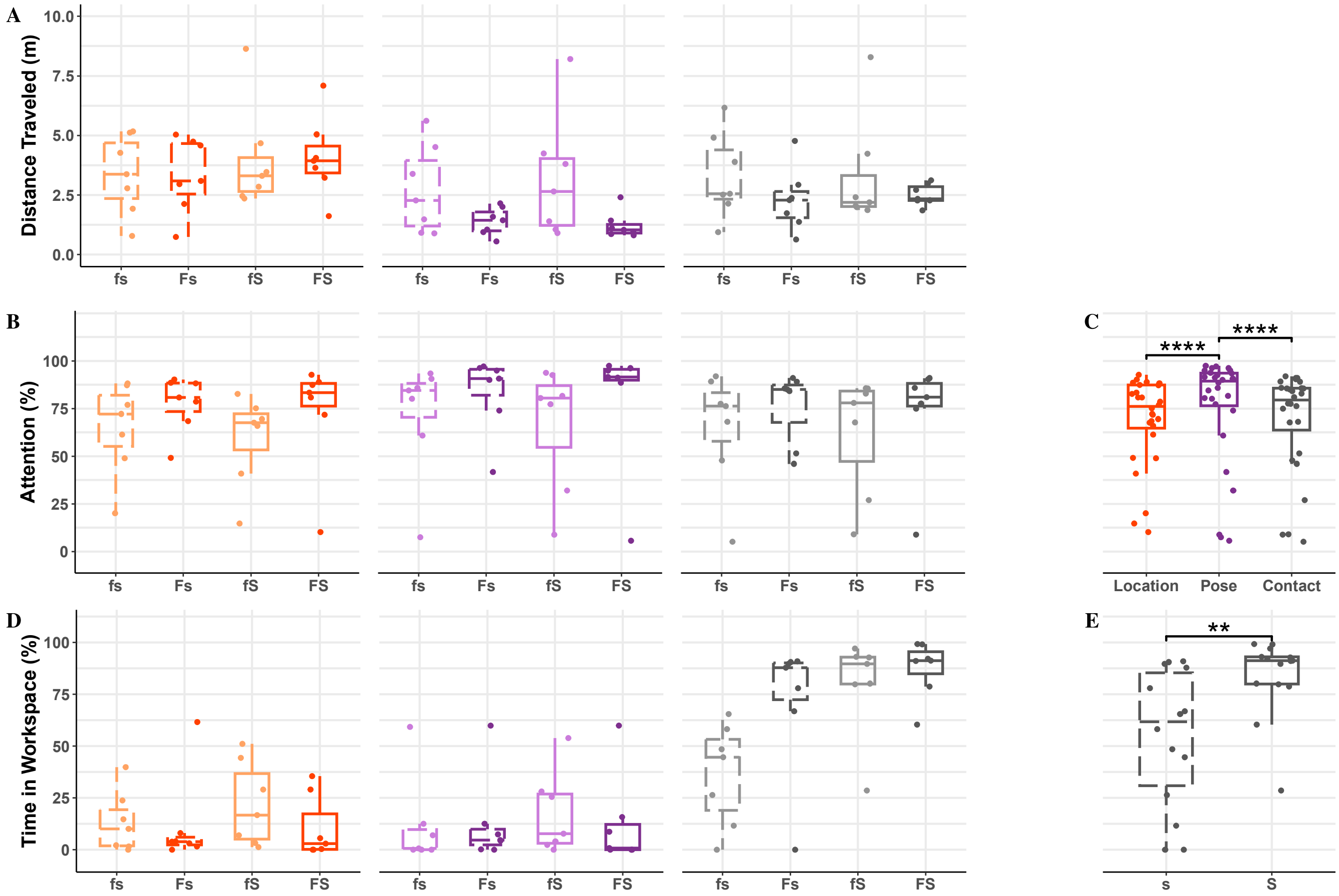

Figure S9. **(A)** Mean distance traveled by each participant for each type of cue, organized by the four feedback conditions (fs, Fs, fS, and FS). **(B)** Mean attention level that the user paid to the robot. **(C)** Users paid significantly more attention to the robot during the pose cue compared to the location and contact cues. **(D)** Mean percentage of trial time that the participants spent within the workspace of the robot. **(E)** Summative feedback significantly increased the time participants spent in the robot's workspace for the contact cue. Though formative feedback also had a significant interaction with cue type for this metric, post-hoc results revealed no interesting observations.

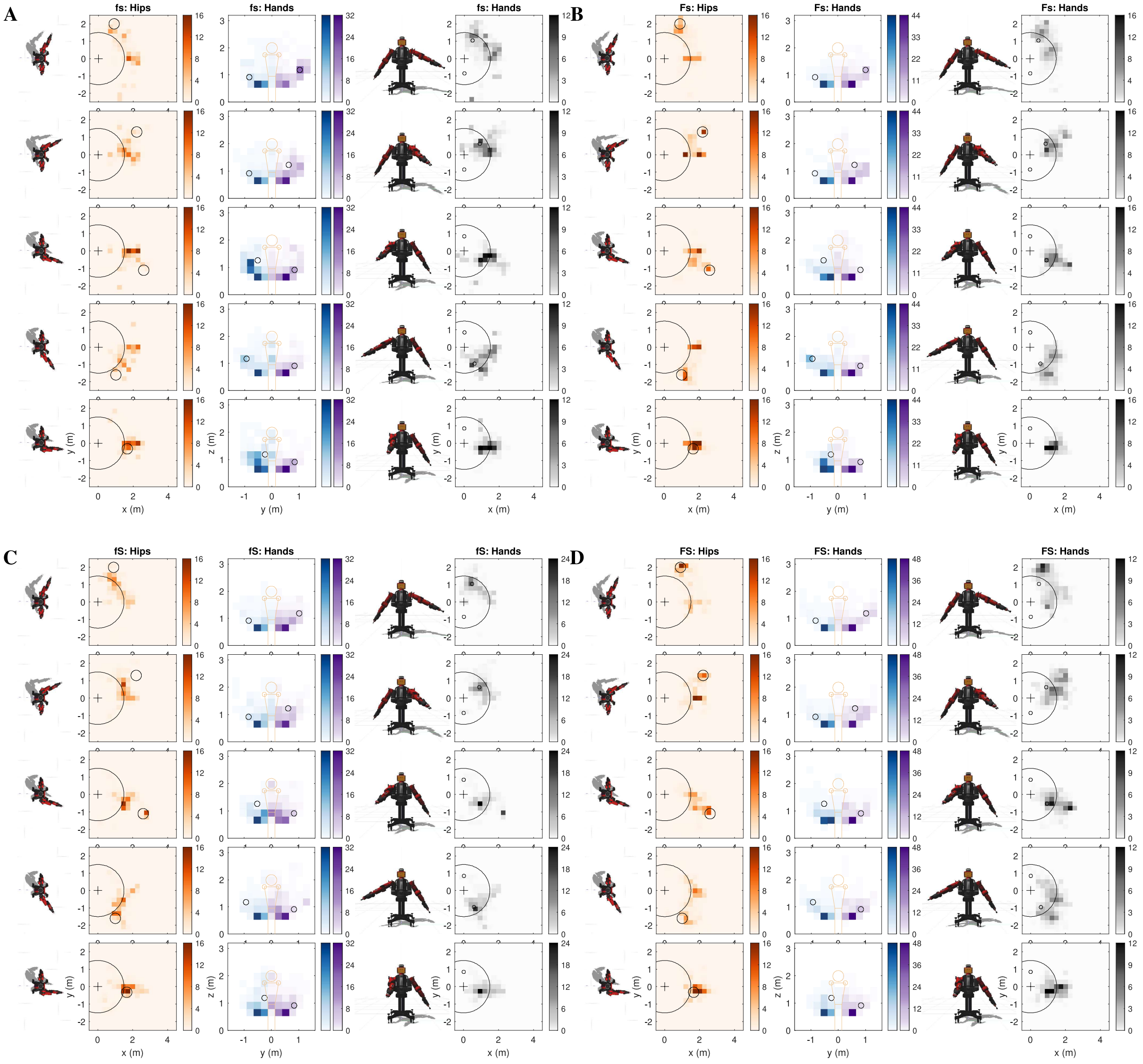

Figure S10. Heatmap visualizations of participant activities for the location cue, separated into the four feedback conditions. Each subfigure comprises three columns of heatmaps. The first column represents the top view of the user position, which enables one to see how much time was spent by the user in different positions of the room. The second column provides an overview of the gesture performed by the user via a front view of the user's right and left hands with respect to their body. Finally, the last column represents a top view of the position of the user hand that was closest to the hand that the robot offered. If both arms of the robot were offered, this heatmap depicts both hands of the user. **(A)** shows the condition where no feedback was provided (fs). **(B)** is the case where only formative feedback was provided (Fs). **(C)** depicts summative feedback only (fS). **(D)** shows both types of feedback (FS).

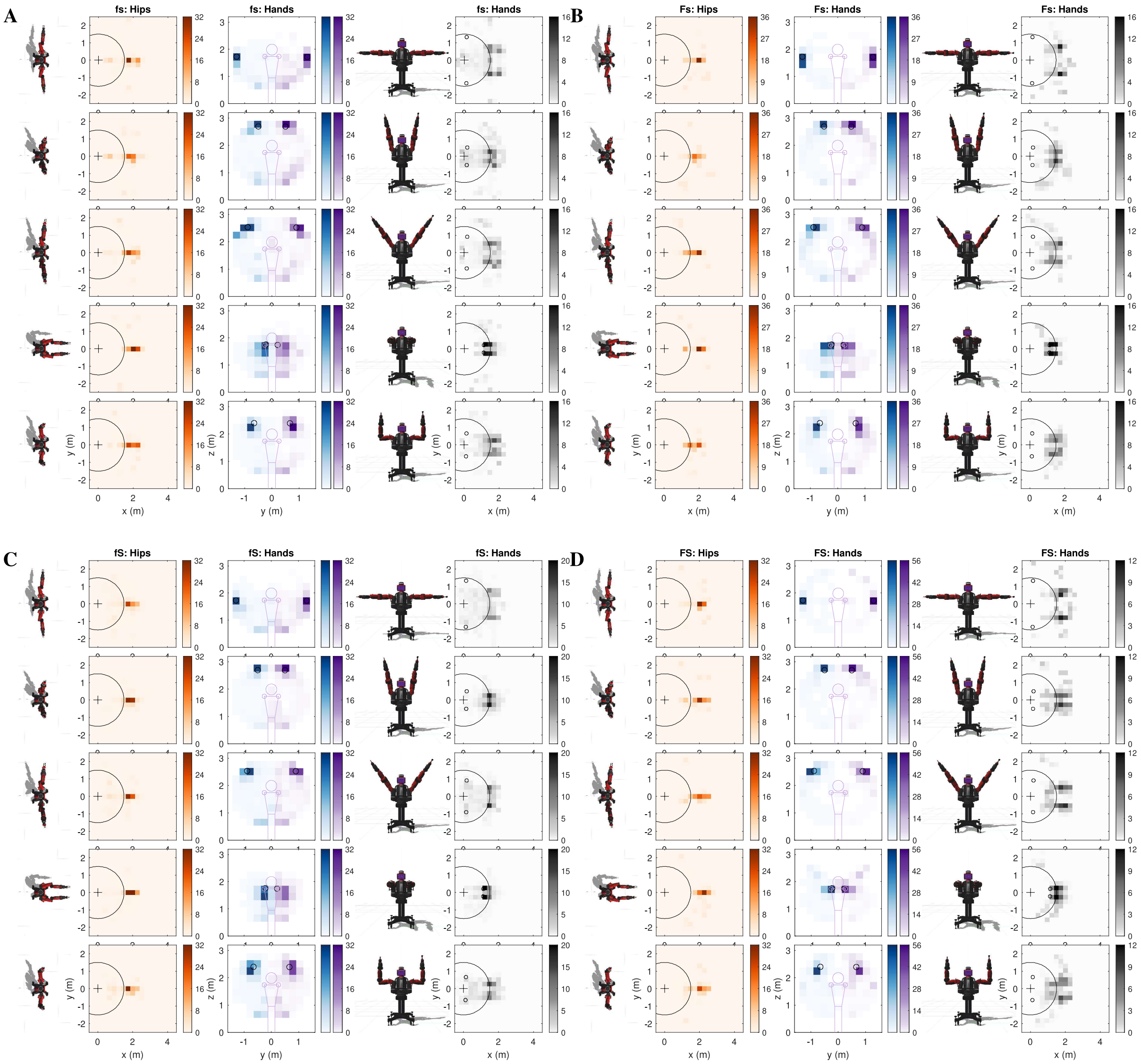

Figure S11. Heatmap visualizations of participant activities for the pose cue, separated into the four feedback conditions. All details are the same as in Fig. S10.

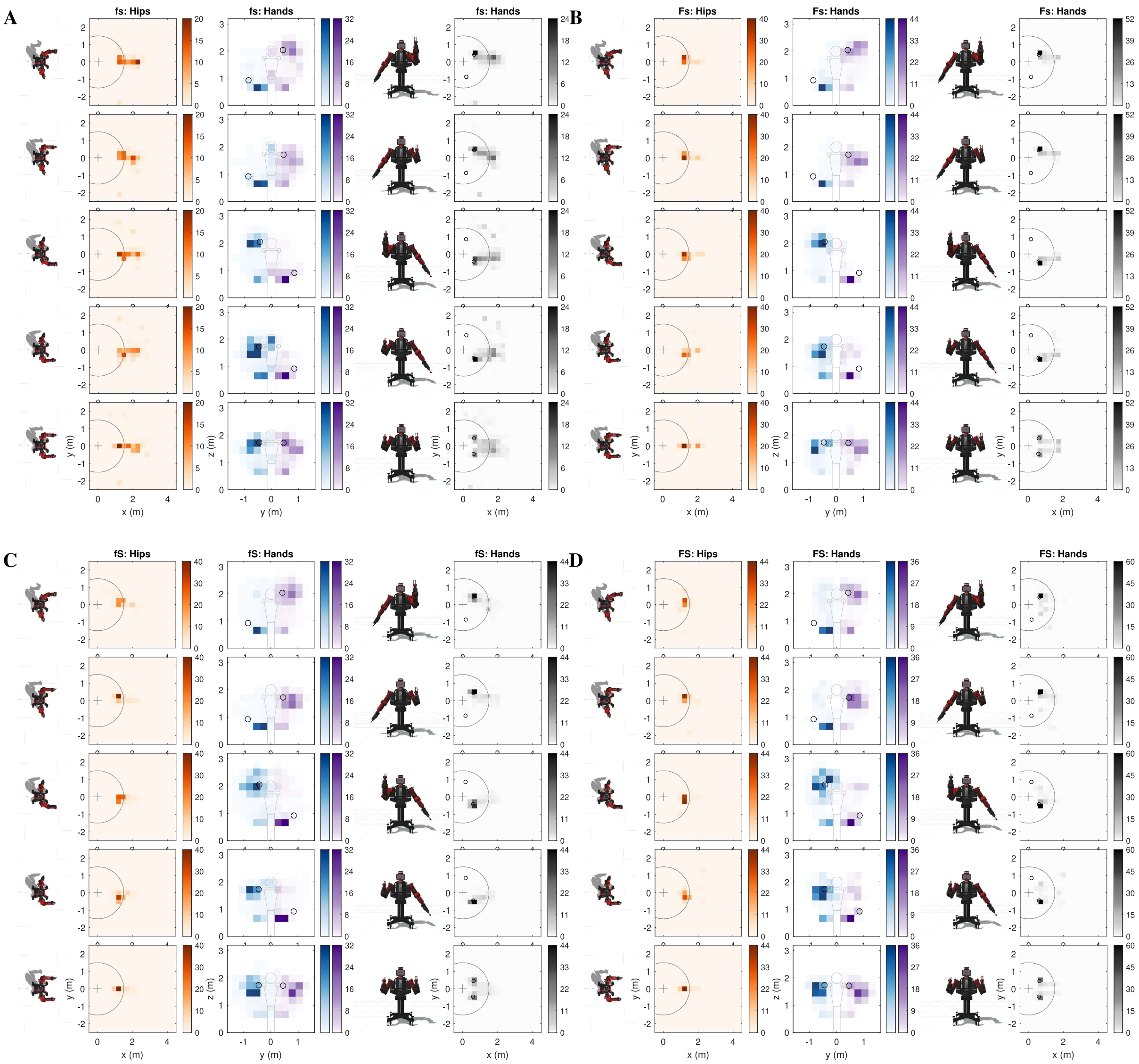

Figure S12. Heatmap visualizations of participant activities for the contact cue, separated into the four feedback conditions. All details are the same as in Fig. S10.

Finally, the error metric for the contact cue (ε_c) at time t was defined as the 3D Euclidean distance from the center of the offered robot hand ($\vec{b}_c$) to the center of the user's closest hand in 3D space. Assuming that $\vec{u}_R(t)$ and $\vec{u}_L(t)$ are the time-varying positions of the user's right and left hands, respectively, ε_c can be calculated as follows:

$$\varepsilon_c(t) = \min\left(\left|\vec{b}_c - \vec{u}_L(t)\right|, \left|\vec{b}_c - \vec{u}_R(t)\right|\right) \tag{5}$$

A special case of the contact cue occurs in the fifth variant when the robot offers both hands to the user. Here, $\vec{b}_R$ and $\vec{b}_L$ are the right and left robot hand positions, defined to be at each parallel-jaw gripper's center. To enforce that both hands of the user should touch the robot hands, ε_c is defined as the maximum of the minimum distances from each user hand to either robot hand, as follows:

$$\varepsilon_c(t) = \max\left(\min\left(\left|\vec{b}_L - \vec{u}_R(t)\right|, \left|\vec{b}_R - \vec{u}_R(t)\right|\right), \min\left(\left|\vec{b}_L - \vec{u}_L(t)\right|, \left|\vec{b}_R - \vec{u}_L(t)\right|\right)\right) \tag{6}$$

Each minimum distance must separately fall below the error threshold ($\bar{\varepsilon}_c = 0.1\,\text{m}$), i.e., each user hand must be near one of the robot's hands.

10 Minimum, Mean, and Maximum Error Per Trial

To provide more insights into how formative and summative feedback affected user performance in the study, we calculated and plotted the minimum, average, and maximum instantaneous error (distance from the desired behavior, measured in meters) that occurred in each trial in the study. We then calculated the average of each of these metrics for each user and each cue type, and we plotted the resulting data by feedback condition, as seen in Fig. S13. The minimum error is particularly interesting because it shows whether the user ever did the desired behavior, even if they did not do it long enough to achieve a non-negligible score. Similarly, the maximum error typically occurred at the start of the trial; reducing the maximum error shows that participants were able to move toward performing the desired action before the trial even began, by correctly reading the robot's cue.

Location cue

We did not find any significant differences between the feedback types for minimum, mean, or maximum error (Fig. S13A) for the location cue. Visual inspection indicates that feedback might have slightly reduced the errors, but these trends are not statistically significant.

Pose cue

As shown in Fig. S13B, we evaluated the effects that both types of feedback had on the errors for the pose cue. First, there was a statistically significant main effect of formative feedback on the minimum error ($F(1,24) = 14, p = 0.00093, \eta_p^2 = 0.37$), mean error ($F(1,24) = 7.9, p = 0.0098, \eta_p^2 = 0.25$), and maximum error ($F(1,24) = 12, p = 0.0021, \eta_p^2 = 0.33$). The presence of formative feedback reduced all three types of errors: minimum ($t(24) = 3.8, p = 0.00093$), mean ($t(24) = 2.8, p = 0.0098$), and maximum ($t(24) = 3.5, p = 0.0021$). Second, summative feedback also had a significant effect on the minimum error ($F(1,24) = 8.6, p = 0.0073, \eta_p^2 = 0.26$) but had no effect on the mean or maximum errors. Specifically, the presence of summative feedback led to a decrease in minimum error ($t(24) = 2.9, p = 0.0073$). Thus, both types of feedback reduced user error in the pose cue.

Contact cue

Finally, we evaluated the effects that both types of feedback had on the errors for the contact cue (Fig. S13C). There was a significant two-way interaction between formative and summative feedback for mean error ($F(1,24) = 5.4, p = 0.029, \eta_p^2 = 0.18$). The presence of only summative feedback ($t(24) = 3, p = 0.04$) or both types of feedback ($t(24) = 3.4, p = 0.016$) significantly reduced the error compared to when no feedback was provided. Furthermore, there was a statistically significant main effect of summative feedback on the minimum error ($F(1,24) = 4.5, p = 0.044, \eta_p^2 = 0.16$); the presence of summative feedback reduced minimum user errors for the contact cue ($t(24) = 2.1, p = 0.044$). However, only formative feedback had a significant effect on maximum error ($F(1,24) = 7.8, p = 0.01, \eta_p^2 = 0.25$); the presence of formative feedback reduced the maximum error for the contact cue ($t(24) = 2.8, p = 0.01$). Thus, we see that both types of feedback significantly reduced some aspects of user error for this cue.

11 Additional Score Analyses

We looked at the mean time it took participants to first perform the desired action in a trial (Fig. S14). If a participant did not perform the solution to a cue within a trial, this metric was set to 30.1 s so that it is higher than all other possible values. A three-way ART ANOVA revealed that there were no significant interaction effects. However, there were significant main effects of

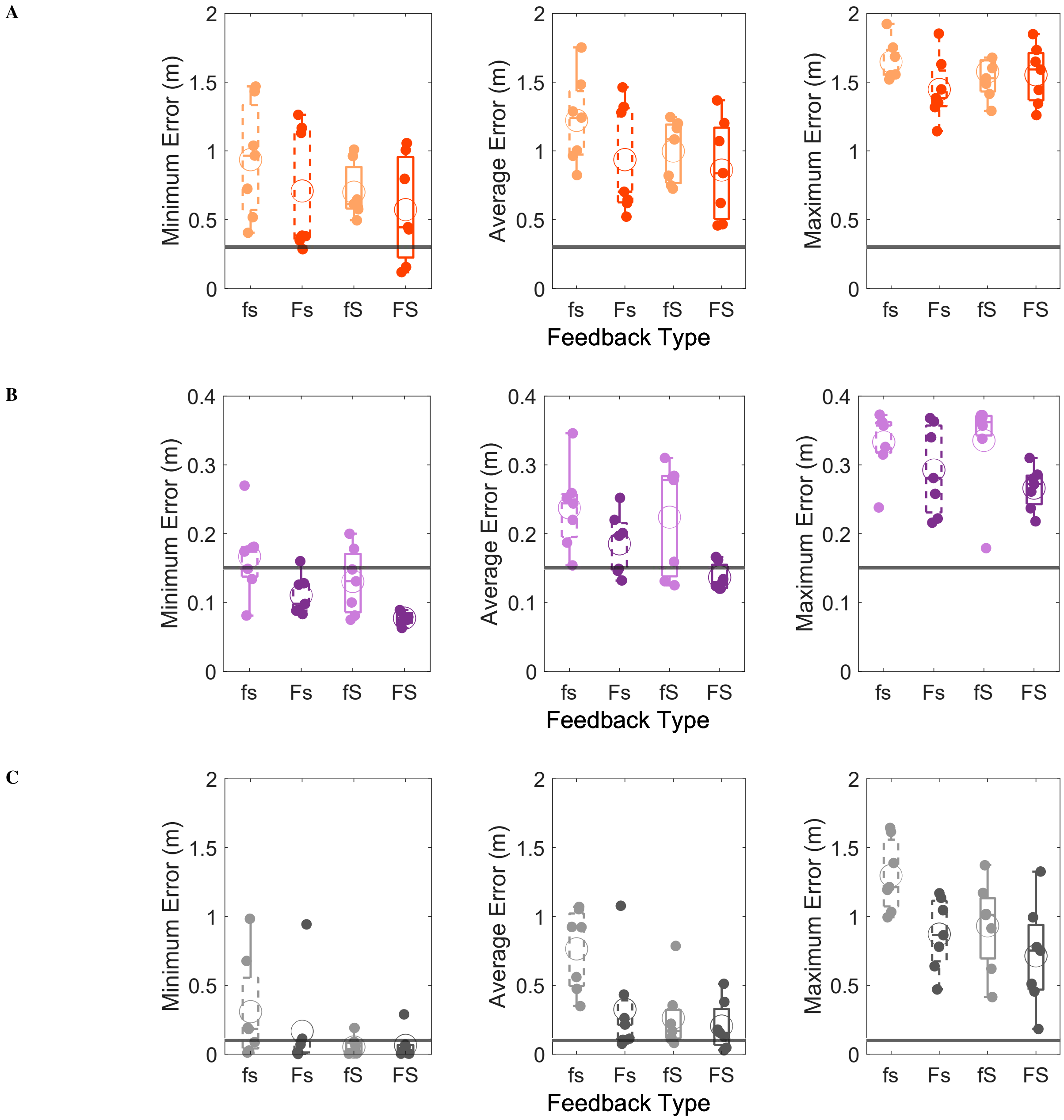

Figure S13. Minimum, average, and maximum instantaneous errors for each cue across feedback conditions. Lower errors are better. The gray line indicates the threshold used to determine whether the user was performing the task correctly. **(A)** Errors for the location cue. There are no significant differences for this cue type. **(B)** Errors for the pose cue. The presence of formative feedback significantly reduced the minimum, average, and maximum errors, while summative feedback significantly reduced maximum error. **(C)** Errors for the contact cue. Interestingly, summative feedback significantly reduced minimum error, whereas formative feedback reduced maximum error.

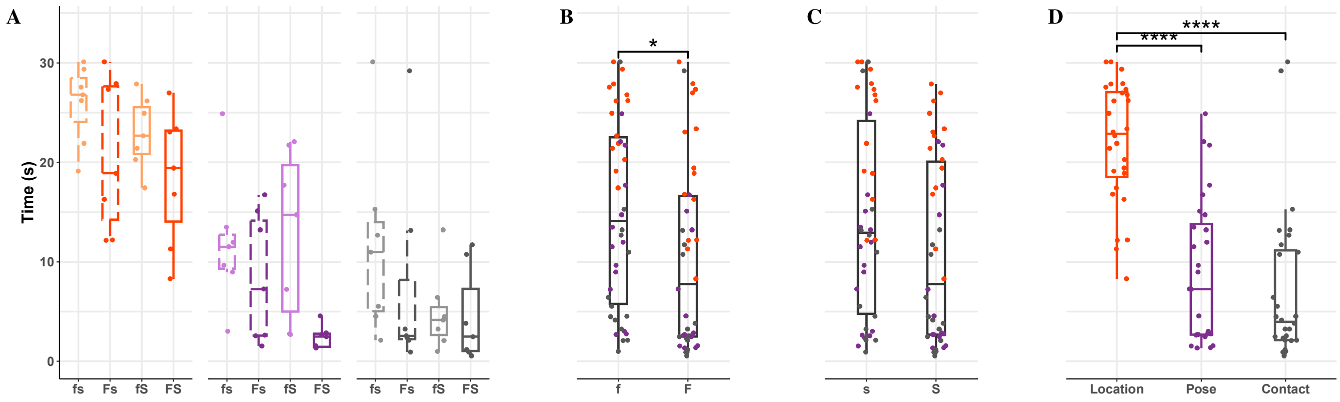

Figure S14. **(A)** Mean time taken by a participant to identify the desired action for a cue in a trial. **(B)** Mean time taken separated by level of formative feedback (fourteen participants in each formative feedback level with responses for all three cue types). Formative feedback significantly improved how quickly participants performed the desired action within a trial. **(C)** Mean time taken separated by level of summative feedback (fourteen participants in each summative feedback level with responses for all three cue types). Though summative feedback did not have a statistically significant impact, it led to a reduction in this metric. **(D)** Mean time taken separated by cue type (all 28 participants for each cue type). Participants performed the desired action for the pose and contact cues more quickly than the location cue. Significance notation: $\star$ $p < 0.05$ and $\star\star\star\star$ $p < 0.0001$.

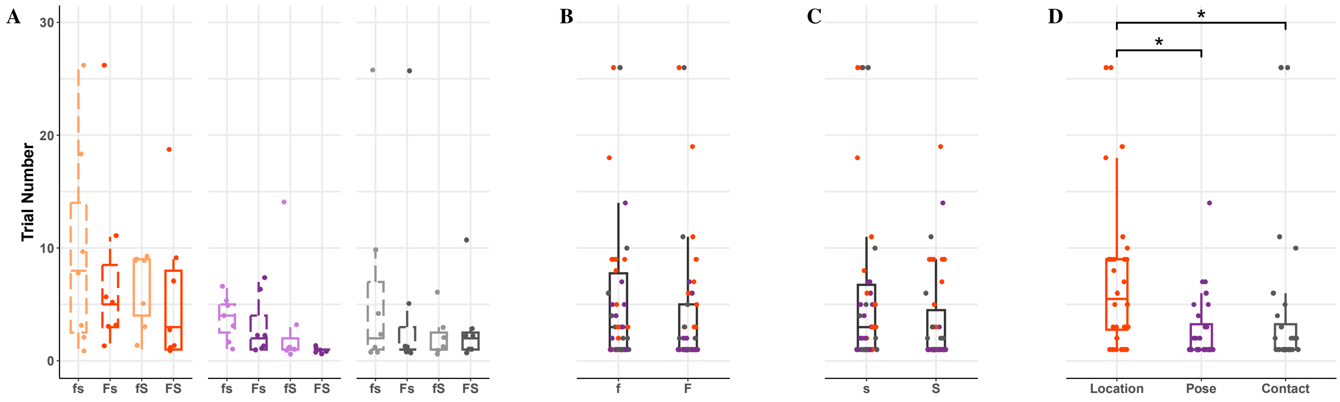

Figure S15. **(A)** First trial where the participant obtained a score in the medium summative category. **(B)** First trial where participants obtained a medium summative score separated by level of formative feedback. Formative feedback significantly improved how quickly participants obtained a better score. **(C)** First trial where participants obtained a medium summative score separated by level of summative feedback. Though summative feedback did not have a statistically significant impact on this metric, it led to an improvement in participants' performance. **(D)** First trial where participants obtained a medium summative score separated by cue type. Participants figured out the solution for the pose and contact cues better than the location cue. Significance notation: $\star$ $p < 0.05$.

formative feedback ($F(1,24) = 7.4, p = 0.012, \eta_p^2 = 0.24$) and cue type ($F(2,48) = 46, p = 6.6 \times 10^{-12}, \eta_p^2 = 0.66$). Within a trial, participants performed the desired action faster if formative feedback (Fig. S14B) was present ($t(24) = 2.7, p = 0.012, d = 0.82$). Though not significant, summative feedback also helped participants identify the solution faster (Fig. S14C). They also identified the solution quicker for the pose ($t(48) = 7.5, p = 4.3 \times 10^{-9}, d = 2$) and contact ($t(48) = 9, p = 2.4 \times 10^{-11}, d = 2.4$) cues when compared to the location cue (Fig. S14D).

Additionally, we looked at two other metrics to evaluate participant learning over time. The first of these metrics reports the first trial number in which the participant achieved a score that falls in or above the medium (yellow) summative category, denoting scores greater than or equal to 10% of the maximum (Score $\geq$ 3 s). The second metric records the first trial number where the participant attained a score in the high (green) summative category, which means they achieved a score greater than or equal to 60% (Score $\geq$ 18 s). In instances where participants never once performed the solution to a cue, these metrics were set to 26 to exceed the actual number of trials.

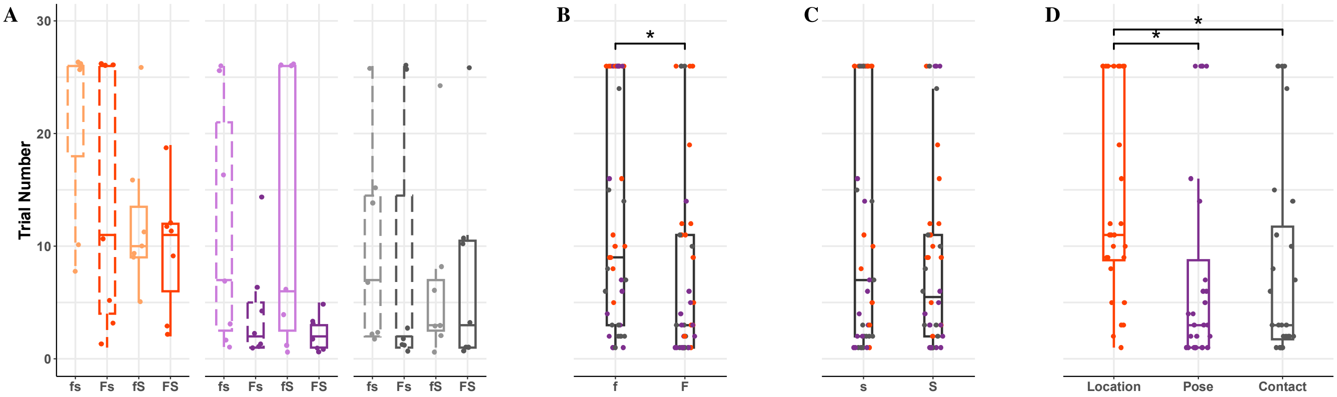

Figure S16. **(A)** First trial where the participant obtained a score in the high summative category. **(B)** First trial where participants obtained a high summative score separated by level of formative feedback. Formative feedback significantly improved how quickly participants obtained an excellent score. **(C)** First trial where participants obtained a high summative score separated by level of summative feedback. Though summative feedback did not have a statistically significant impact on this metric, it led to an improvement in participants' performance. **(D)** First trial where participants obtained a high summative score separated by cue type. Participants figured out the solution for the pose and contact cues better than the location cue. Significance notation: $\star\ p < 0.05$.

For the medium summative score category, there were no significant interaction effects or main effects of either type of feedback (Fig. S15A). Though not significant, both types of cues led to participants identifying the solution earlier in the progression of the study (Fig. S15B and Fig. S15C). There was, however, a statistically significant main effect of the type of cue $(F(2,48) = 5.2, p = 0.0089, \eta_p^2 = 0.18)$. Post-hoc tests revealed that this metric was lower for the pose $(t(48) = 3, p = 0.012, d = 0.82)$ and contact cues $(t(48) = 2.5, p = 0.046, d = 0.68)$ when compared to the location cues (Fig. S15D).

For the high summative score category, the three-way ART ANOVA revealed no statistically significant interaction effects (Fig. S16A). However, the main effects of formative feedback $(F(1,24) = 6.3, p = 0.019, \eta_p^2 = 0.21)$ and cue type $(F(2,48) = 5.4, p = 0.0076, \eta_p^2 = 0.18)$ were significant. Participants were able to obtain a higher trial score earlier in the study if formative feedback was present $(t(24) = 2.5, p = 0.019, d = 0.53)$ (Fig. S16B). Though not significant, the presence of summative feedback also led to a faster increase in scores (Fig. S16C). Participants were able to earn a higher score earlier for the pose $(t(48) = 2.8, p = 0.025, d = 0.74)$ and contact $(t(48) = 2.9, p = 0.016, d = 0.78)$ cues when compared to the location (Fig. S16D).

12 Legends for Videos

Participants shown in the videos explicitly provided informed consent to sharing these videos as a part of publications associated with this research study.

Legend for Video SV1: This video (with sound) shows an example of how the robot moved when presenting each task cue. These instructions were shown at the start of each trial and remained the same for all four feedback conditions (fs, Fs, fS and FS). Zoomed-in and silent versions of these videos were shown to the users after the study as a part of the cue evaluation questions (Table S4).

Legend for Video SV2: This video (with sound) shows a set of six back-to-back trials for a study participant who experienced both formative and summative feedback (FS). All three high-resolution camera views from around the Robot Interaction Studio[64] are shown to facilitate 3D understanding of the movements that occurred. The participant wears a heart-rate monitor around their left bicep and a wireless lapel microphone for the audio recording of the study. This set of trials was chosen based on three major criteria. The first was to demonstrate at least one example of formative feedback for each task cue. The second criterion was to show at least one example of each type of summative feedback (red, yellow, and green). Finally, at least two different variants of each task cue can be seen in the video. In addition, we have incorporated overlaid annotations to illustrate how the robot's face changes in response to participant actions. Given the noisy nature of the audio data from the study, we have also overlaid the sounds produced by the robot at the beginning of each cue and when it provides summative feedback. Please note that the annotations were done manually and may include slight human error.

The depicted participant is one of the explorers who eventually identified the solutions to all three task cues. These six trials occurred during the early part of the experiment, specifically trials 13 to 18 of the 75 total trials. Participants generally spent this time exploring robot behaviors. For the location cue, the participant can be seen utilizing the robot's gesture-based feedback and facial expressions to identify the correct location to walk to. This participant previously saw the cue presented in trial 13 but not the one presented in trial 16. For the pose cue, the participant can be seen performing the correct action for both cue variants shown. The participant can also be seen attempting various actions to observe how the robot responds. The participant already saw the cues presented in trials 14 and 17 but still struggles a bit with the cue shown in trial 17. The participant can be seen adjusting his pose to perform the task correctly. The contact cues presented in trials 15 and 18 are both new to the participant. Although they did not identify the correct solution in these two trials, the participant can be seen briefly touching the robot's offered end-effectors, with flashes of positive formative feedback (a happy facial expression); they are thus on the way to figuring out this cue from the formative and summative feedback provided by the robot. There is a short break in the video between trials 15 and 16 due to a quick pause in the recording software.
